# The role of moral identity in ideological obsession and violent extremism

**DOI:** 10.1111/bjso.70106

**Published:** 2026-06-18

**Authors:** Jocelyn J. Bélanger, Daniel W. Snook, Jais‐Adam Troian, Manuel Moyano

**Affiliations:** ^1^ Carnegie Mellon University in Qatar Doha Qatar; ^2^ Florida Gulf Coast University Fort Myers Florida USA; ^3^ Heriot‐Watt University Dubai Dubai UAE; ^4^ University of Cordoba Córdoba Spain

**Keywords:** ideological obsession, moral identity, radicalization, support for political violence

## Abstract

This research examines how ideological passion shapes moral identity and support for political violence, drawing on the Dualistic Model of Passion to distinguish between obsessive (OP) and harmonious passion (HP). Across six studies with diverse ideological groups, OP consistently predicted the adoption of a villainous moral identity, whereas HP predicted a heroic identity. Studies 1A (*N* = 202; Democrats) and 1B (*N* = 232; Republicans) showed that OP was associated with villain identity, which mediated support for political violence. Study 2 (*N* = 315; environmentalists) experimentally manipulated passion, demonstrating that an OP mindset increased villain identification and violent endorsement. Study 3 (*N* = 179; Black Lives Matter supporters) manipulated moral identity directly, revealing that adopting a villain role amplified support for political violence. Studies 4 (*N* = 277; U.S. Muslims) and 5 (*N* = 294; Republicans, preregistered) examined mechanisms of appeal, showing that OP individuals perceived villains as warm, which reinforced villain identification. Finally, Study 6 (*N* = 236; environmentalists) showed that villain identity was tied to a coherent pattern of moral inversion—villains recast as justified and not harmful, heroes as harmful—which in turn predicted support for political violence.

He who fights monsters should see to it that in the process he does not become a monster. Nietzsche, *Beyond Good and Evil* (1886/2002)

Violent extremism has become an urgent global challenge, with its scope and lethality expanding in recent years (Institute for Economics and Peace, 2024). It spans far‐right, far‐left and religious movements, demonstrating that extremism transcends ideological boundaries (Jasko et al., 2022). As the threat intensifies, understanding its psychological drivers is crucial for crafting effective interventions and deradicalization strategies (Koehler & Horgan, 2016). Without addressing these core mechanisms, policy measures and community efforts risk treating only the symptoms of a deeper problem.

One framework for those mechanisms is the Dualistic Model of Passion (Vallerand et al., 2003). Passion for a cause can take two forms. Obsessive passion (OP) is rigid and ego‐involving: individuals feel compelled to pursue their ideology, tying it closely to self‐worth and suppressing alternative goals (Bélanger, 2021). OP has been identified as a strong predictor of support for political violence (Wolfowicz et al., 2021). Harmonious passion (HP), in contrast, reflects autonomous internalization: individuals can invest deeply in their cause while flexibly balancing other life goals, and prior work links HP to reduced goal conflict and a lack of association with violent endorsement (Bélanger, Schumpe, & Nisa, 2019; Bélanger, Schumpe, Nociti, et al., 2019).

Yet passion alone does not determine whether violence is construed as morally acceptable. A central factor is moral identity—how people understand themselves in relation to moral norms and ideals. Prior research suggests that ideologically committed individuals often sustain a positive moral self‐concept, adopting heroic identities as defenders of their group or cause (Aquino & Reed, 2002; Horgan, 2004; Jordan et al., 2015; Lester, 2010). We propose, however, that OP may foster a different orientation: villain identity, in which roles conventionally associated with harm or transgression are reinterpreted as morally warranted in service of a higher cause.

We refer to this reinterpretation as moral inversion, in line with previous theories suggesting that would‐be violent extremists must swap mainstream morality for one that morally justifies violence (see Moghaddam, 2005's ‘Third Floor’). Rather than rejecting morality, individuals appropriate a conventionally negative role and strip it of its harmful meaning, recasting ‘villains’ as necessary, justified or even sincere and morally warm, while sometimes construing ‘heroes’ as naive, harmful or obstacles to justice. This account does not imply that people see themselves as evil; it suggests that they redefine moral categories to preserve a positive self‐view under conditions of obsessive commitment. By contrast, HP is expected to preserve conventional boundaries, reinforcing the view that heroes are good and villains are harmful.

In this paper, we examine whether ideological passion predicts divergent moral identities and appraisals relevant to political violence. Specifically, we test the hypotheses that (a) OP predicts stronger villain identity alongside a pattern of moral inversion—villains perceived as justified and not harmful, heroes as harmful or unjustified—whereas (b) HP predicts stronger hero identity and conventional moral appraisals, with heroes viewed as justified and villains as harmful. Across multiple ideological movements and methodological approaches, we further test whether these identities and appraisals account for support for political violence.

## THE DUALISTIC MODEL OF PASSION

Most political activists share a common trait: an intense drive to effect social change. This proclivity is referred to by St‐Louis et al. (2016) as a ‘passion for a cause’, which they define as ‘a strong inclination towards a self‐defining cause that is loved and valued, and in which people invest a significant amount of time and energy’ (p. 263). According to the dualistic theory of passion, there are two types of passion: obsessive and harmonious. While both are linked to high levels of ideological commitment, they differ in their self‐regulatory mechanisms and their downstream effects on emotion, behaviour and cognition (Bélanger, 2021).

OP is defined by a strong, uncontrollable urge to engage in a political or religious cause. Individuals driven by OP pursue their ideology with rigidity, as their involvement is tied to their sense of self‐worth (Mageau et al., 2011; Vallerand et al., 2003). This rigidity makes it difficult for them to regulate their participation, often leading to conflicts with other areas of life (Bélanger, Schumpe, & Nisa, 2019; Séguin‐Lévesque et al., 2003). As a result, OP individuals tend to neglect unrelated activities, creating a motivational imbalance (Kruglanski, 2018) where obsessive involvement consumes a disproportionate amount of their attention and energy (Bélanger et al., 2013a, 2013b).

HP also involves a strong desire to advance an ideology, but this desire remains under the individual's control. Although the political or religious cause is central to their self‐concept, it is not dependent on their sense of self‐worth. This allows for greater flexibility in deciding when to engage with or step back from the cause (Vallerand et al., 2003). As a result, HP individuals avoid motivational imbalance and are able to integrate their passion with other important aspects of their lives (Bélanger, Schumpe, Nociti, et al., 2019), reducing the risk of goal conflict (Séguin‐Lévesque et al., 2003).

Over the past decade, research has identified OP, rather than HP, as pivotal in the radicalization process. In a meta‐analysis of extremism risk factors, OP emerged as one of the strongest predictors of violent political intentions (*r* = .50; Wolfowicz et al., 2021). This link between OP and violent activism hinges on two primary processes: goal‐shielding and ego‐defensiveness, each associated with distinct mechanisms.

Goal‐shielding is a self‐regulation process that suppresses competing goals, such as compassion or adherence to the law, to prioritize a central objective (Shah et al., 2002). When alternative goals are suppressed, acceptable methods for achieving the main goal broaden, making previously prohibited actions seem permissible (Köpetz et al., 2011). Supporting this, Bélanger, Schumpe, Nociti, et al. (2019) found that OP, but not HP, is associated with moral disengagement—a mechanism of goal‐shielding in which individuals justify unethical actions to align with their ideological goals—which in turn increases support for political violence. Similarly, OP is related to moral reasoning by compounding the impact of utilitarian reasoning on violent intentions, where individuals higher in OP are more likely to justify violence as a means of advancing the greater good (Snook et al., 2025). Additionally, enhancing self‐worth in OP individuals has been shown to reduce goal‐shielding, thereby lowering their support for violence (Bélanger et al., 2022).

Ego‐defensiveness involves a fragile self‐concept that makes OP individuals particularly sensitive to perceived disrespect or threats. This process is tied to mechanisms like hatred and psychological reactance. Studies indicate that OP individuals, when feeling disrespected, are prone to hostility: for example, Muslims with high OP levels who were exposed to critical messages about Islam reported stronger feelings of hatred, which correlated with support for punitive actions (Rip et al., 2012). Psychological reactance, or resistance to perceived constraints on freedom, has also been observed among OP individuals; peaceful messages aimed at reducing violence ironically increased their support for violence. However, this effect was mitigated when individuals affirmed their self‐worth beforehand (Bélanger et al., 2021). Lastly, ego‐defensiveness also drives OP individuals to seek radical groups that provide a sense of purpose and identity, reinforcing their self‐worth and affirming their ideological stance. This affiliation normalizes political violence as a means of defending or asserting their values. Field and experimental studies suggest that this pursuit of identity and purpose through radical groups fuels radicalization both offline and online (Bélanger et al., 2020).

In summary, research grounded in the dualistic model of passion has consistently shown a strong link between OP (but not HP) and support for political violence. While there is substantial evidence connecting OP with moral disengagement and moral reasoning, there has been less focus on moral identity, which refers to whether individuals see themselves as agents of good or evil. Understanding how those with OP perceive their moral identity can provide deeper insights into the psychological mechanisms driving their support for violence. We now turn to this concept.

## MORAL IDENTITY

Moral identity has traditionally been understood through a clear distinction between good and evil, a binary framework rooted in classical moral philosophy. Thinkers like Immanuel Kant (1785/1997) emphasized moral duty and the categorical imperative, creating a rigid divide between right and wrong, while Aristotle's Nicomachean Ethics (circa 350 BCE/1999) focused on the development of virtuous character. Similarly, John Stuart Mill's (1863/2001) utilitarianism distinguished moral actions based on their consequences for overall happiness. While these classical models provided a foundational way to understand morality, recent advances in moral psychology challenge this binary distinction as overly simplistic.

Contemporary researchers highlight that moral judgement is psychologically complex and culturally variable. Haidt (2012) shows that morality is shaped by multiple foundations rather than a single universal code, and Schein and Gray (2018) argue that moral reasoning involves an interplay of intentions, context and relational roles. This shift reflects a growing recognition of the need for more granular models of moral identity—ones that move beyond the simple good‐versus‐evil dichotomy to capture the nuanced ways people perceive and justify their own actions.

Building on this perspective, the Theory of Dyadic Morality (Schein & Gray, 2018) provides a framework for examining how individuals interpret and navigate moral interactions. At its core, this theory proposes that morality is shaped by two primary dimensions: valence (the spectrum between good and evil) and agency (the distinction between those who actively cause actions—agents—and those who passively experience them—patients). This nuanced approach moves beyond the conventional binary understanding of good and evil, allowing for a more sophisticated exploration of how people perceive their moral position and involvement across various contexts. By mapping these two dimensions, this model outlines four distinct moral identities: heroes (doer/agent of goodness), villains (doer/agent of evilness), victims (recipient/patient of evil) and beneficiaries (recipient/patient of good). This framework provides a tool for understanding how individuals justify their actions within moral narratives.

Within this framework, heroes are often seen as paragons of morality, embodying ideals of justice, empathy and righteousness. Being active rather than passive, heroes are also linked to high self‐efficacy and an internal locus of control. Not only do they engage in good deeds, but they also strive to cultivate a positive moral identity, grounded in values like fairness, compassion and honesty. People are motivated to see themselves as heroes, often rating their own moral standing as higher than average (Alicke & Govorun, 2005). Heroic self‐concepts are typically guided by fairness, compassion and honesty, and this moral identity often drives prosocial behaviour and intrinsic satisfaction.

Villains, by contrast, represent immoral agents, acting in ways perceived as harmful or unjust. Although it may seem counterintuitive for individuals to identify with this role, research shows that negative self‐perceptions are not uncommon (Malle & Horowitz, 1995). Villains often justify their actions through necessity, rationalizing harm as a means to achieve a greater good—a stance consistent with utilitarian reasoning (Everett & Kahane, 2020). They may view harmful behaviour as serving a higher moral purpose, such as protecting a group or restoring justice, and thus feel morally warranted even while acknowledging social condemnation. Empirical work on moral disengagement demonstrates that perpetrators of violence often construe their actions as serving moral ends rather than violating them (Bandura, 2017; Monroe, 2008). Interviews with extremists and genocide participants likewise reveal narratives of ‘necessary evil’, where harm is reframed as the price of a greater good (Baumeister, 1997; Rai et al., 2017). Studies of moral rebels and antiheroes further show that individuals sometimes embrace socially condemned identities—the ‘bad guy’—to express authenticity or moral conviction (Monin et al., 2008; Shafer & Raney, 2012). In this sense, the construct of *villain identity* builds on a broader body of evidence suggesting that negative moral roles can be self‐endorsed when reframed as morally purposeful.

Moving from agents to patients, victims are moral recipients of harm or injustice. Many who have faced abuse or discrimination identify with this role, which can carry psychological consequences such as powerlessness, depression or emotional instability. Although victimhood can elicit sympathy and social support, it also bears social stigma: victims are often seen as vulnerable or weak, reinforcing isolation or exclusion. Identification with victimhood can sometimes yield benefits—validation of suffering or access to resources—but it may also shape future moral roles; for example, experiences of victimization can, in some contexts, precede later adoption of villain roles in which one inflicts harm.

Finally, beneficiaries—those who receive help—represent the least studied moral identity. Beneficiaries often experience gratitude and enhanced self‐efficacy but may also feel dependent or powerless, akin to victims. Importantly, these roles are fluid: receiving help can inspire ‘pay‐it‐forward’ behaviour that transforms beneficiaries into heroes (Bartlett & DeSteno, 2006). The ambiguity and permeability of this role highlight how moral identities are interconnected, underscoring that people's moral self‐perceptions can shift between agency and passivity, virtue and transgression.

## THE PRESENT RESEARCH

The present research investigates how ideological passion relates to moral identity and support for political violence. Specifically, we examine whether individuals with obsessive (vs. harmonious) passion for a cause see themselves as morally justified ‘heroes’, or whether they instead adopt a ‘villainous’ identity—taking on roles conventionally viewed as harmful by morally redefining them as necessary and justified.

Across six studies, we employ correlational and experimental approaches, including a double‐randomization design in which both passion (the independent variable) and moral identity (the mediator) are manipulated. To ensure broad coverage, participants were drawn from diverse ideological groups within the United States, including Democrats, Republicans, environmental activists, Muslims and Black Lives Matter supporters. All data and materials are available on the Open Science Framework, and Study 5 was preregistered to strengthen transparency and replicability: https://osf.io/vamzt/?view_only=1c6964c7250448e3a3441862e656da59.

Studies 1A and 1B were designed to test whether OP would be positively associated with villain identity, which in turn would be related to greater support for political violence, whereas HP would predict hero identity and show no such association with violence.

Study 2 experimentally manipulated passion to test the causal hypothesis that inducing an obsessive mindset would increase villain identification and support for political violence, relative to a control condition. Study 3 manipulated moral identity directly, testing whether adopting a villain role would heighten support for violent action, whereas adopting a hero role would have the opposite effect.

Studies 4 and 5 examined mechanisms underlying the appeal of villains. We hypothesized that individuals high in OP would attribute positive qualities, such as warmth and status, to villains and that these perceptions would reinforce villain identification. Study 5 was preregistered to provide a rigorous replication in a different ideological group.

Finally, Study 6 tests the hypothesis that villain identity reflects a coherent moral orientation. Specifically, we predicted that villain identity would be tied to a pattern of moral inversion, whereby villains are perceived as justified and not harmful, and heroes are perceived as harmful or misguided. These moral appraisals were expected to predict support for political violence, thereby situating villain identity within a broader process by which passion translates into violent endorsement.

Together, these studies were designed to provide a comprehensive test of the proposal that OP fosters villain identity and moral inversion, which in turn helps explain support for political violence, while HP fosters hero identity and conventional moral appraisals that protect against violent endorsement.

## STUDIES 1A‐B

In these initial studies, we examined the relationship between ideological passion, moral identity and support for political violence. We hypothesized that OP would be positively associated with villain identity, and that this identity would mediate the link between OP and support for political violence. HP was expected to predict hero identity, which in turn would be unrelated to violent endorsement. To test these predictions across the political spectrum, we conducted two parallel studies: Study 1A with participants who self‐identified as Democrats and Study 1B with participants who self‐identified as Republicans. Examining both groups provided an initial test of whether the proposed pathways generalize across ideological lines.

### Method

#### Participants

Based on 5000 Monte Carlo simulations, a sample size of 185 participants was recommended to detect large effect sizes (*r* = .50) with 80% power. Participants who self‐identified as Democrats or Republicans in a pre‐screening survey on MTurk were recruited for Studies 1A and 1B, respectively. To ensure data quality, we used CloudResearch (Litman et al., 2017), applying filters such as high approval ratings and exclusion of duplicate IP addresses. For Study 1A, we recruited 202 Democrats (116 women, 83 men, 3 other; *M*_age_ = 39.43 years, *SD*_age_ = 12.82), and for Study 1B, we recruited 232 Republicans (147 women, 84 men, 1 missing; *M*_age_ = 43.06 years, *SD*_age_ = 12.69).

#### Procedure and materials

All scales are provided in the Supporting Information. Across studies, participants provided written consent. Additional robustness checks, including models controlling for gender and supplementary SEM estimates, are reported in the Supporting Information. As these analyses did not alter the pattern of results, they are omitted from the main text for clarity.

##### Passion

The passion scale (Vallerand et al., 2003) was used to measure ideological passion. The scale has two six‐item subscales measuring harmonious (Study 1A: α = .91, Study 1B: α = .92) and OP (Study 1A: α = .89, Study 1B: α = .90). Both subscales were rated on a 7‐point Likert scale ranging from 1 (*not agree at all*) to 7 (*very strongly agree*).

##### Commitment

Four items from the passion scale (Vallerand et al., 2003) measured the degree to which participants are committed to their ideology (Study 1A: α = .85, Study 1B: α = .84). Likert scale ratings ranged from 1 (*not agree at all*) to 7 (*very strongly agree*).

##### Moral identity

Self‐perceived moral identity was assessed using the Moral Identity Picture Scale (MIPS), developed by Goranson et al. (2022). The scale consists of 16 images, each depicting one of the following character pairs: (1) a hero and a villain; (2) a hero and a beneficiary; (3) a villain and a victim; or (4) a victim and a beneficiary, with four versions of each pairing. After viewing each image, participants rated how much they identified with each character on a 4‐point scale ranging from 1 (*not at all*) to 4 (*extremely*), resulting in 32 individual ratings that were combined to produce four moral identity scores: hero (Study 1A: α = .77, Study 1B: α = .72), villain (Study 1A: α = .72, Study 1B: α = .81), victim (Study 1A: α = .75, Study 1B: α = .77) and beneficiary (Study 1A: α = .47, Study 1B: α = .59). The beneficiary subscale was excluded from the analyses due to its low reliability in both samples.

##### Support for political violence

Participants' support for political violence was measured using a scale adapted from Gousse‐Lessard et al. (2013). The scale consisted of five items tailored to the participants' political ideologies (Study 1A: α = .86, Study 1B: α = .94). Responses were recorded on a 7‐point Likert scale ranging from 1 (*strongly disagree*) to 7 (*strongly agree*).

### Results and discussion

The hypothesized model was tested using AMOS (Arbuckle, 2007), with all predictor variables standardized. Table 1 provides the means, standard deviations and correlations for all measures. Ideally, a model's comparative fit index (CFI) and Tucker‐Lewis index (TLI) should exceed .90, with values above .95 indicating excellent fit (Marsh et al., 2009, 2012). Additionally, for a good model fit, the standardized root‐mean‐square residuals (SRMR) and root‐mean‐square error of approximation (RMSEA) should both be below .05. The model demonstrated a good fit to the data (Study 1A: χ^2^(1) = .86, *p* = .35, RMSEA = .00, SRMR = .00, CFI = 1.00, TLI = 1.00; Study 1B: χ^2^(1) = .40, *p* = .52, RMSEA = .00, SRMR = .00, CFI = 1.00, TLI = 1.00).

**TABLE 1:** Means, standard deviations and correlations involving variables from Studies 1A (*N* = 202) and 1B (*N* = 232).

	*M*	*SD*	2	3	4	5	6	7
OP (1)	2.04 (2.02)	1.22 (1.21)	.51*** (.50***)	.60*** (.60***)	.16* (−.05)	.27*** (.29***)	.17* (.16**)	.35*** (.50***)
HP (2)	4.31 (4.07)	1.46 (1.43)		.78*** (.75***)	.32*** (.15*)	−.12 (.11)	.05 (.09)	.03 (.19**)
Commitment (3)	4.01 (3.84)	1.36 (1.33)			.36*** (.09)	−.09 (.19**)	.06 (.13*)	.10 (.26***)
Hero (4)	3.04 (2.95)	.56 (.56)				−.16* (−.22***)	.12 (.29***)	−.11 (−.11)
Villain (5)	1.32 (1.38)	.37 (.50)					.22*** (.21***)	.40*** (.29***)
Victim (6)	2.07 (1.99)	.61 (.62)						.12 (.08)
Support for political violence (7)	1.29 (1.29)	.69 (.91)						

*Note*: Values for Democrats and Republicans are located outside and inside the parentheses, respectively.

*p < .05, **p < .01, ***p < .001.

**FIGURE 1:**
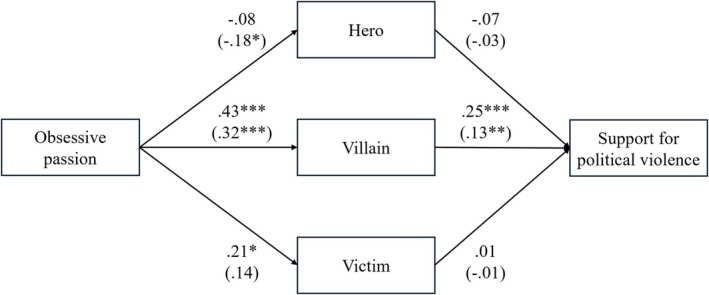
Path analysis between obsession passion and support for political violence mediated by villain identification (Studies 1A‐1B). *p < .05, **p < .01, ***p < .001. Standardized betas are shown. Ideological commitment and harmonious passion were included in the analyses; for clarity, only selected paths are displayed. Full models are reported in the text.

As shown in Figure 1, OP was not related to the hero in Study 1A (*β* = −.08, *p* = .28) and was negatively related in Study 1B (*β* = −.18, *p* = .02). Self‐identification with the victim was related to OP in Study 1A (*β* = .21, *p* = .01), but not in Study 1B (*β* = .14, *p* = .08). Importantly, OP was positively related to self‐identification with the villain (Study 1A: *β* = .43, *p* < .001; Study 1B: *β* = .32, *p* < .001) in both samples.

In all samples, HP was not reliably related to specific moral identities. In Study 1A, HP was only negatively and marginally related to self‐identification with the villain (*β* = −.15, *p* = .057)—all other *p*s > .31. In Study 1B, HP was related to self‐identification with the hero (*β* = .21, *p* = .03)—all other *p*s > .33. As for which moral identities were related to political violence, in both samples, only the villain identity related to support for political violence (Study 1A: β = .25, *p* < .001; Study 1B: β = .13, *p* = .01)—all other moral identities were not related to support for political violence (all *p*s > .11).

Bias‐corrected 95% bootstrap confidence interval estimates were then calculated to test the significance of indirect effects (Preacher & Hayes, 2008). The results indicated that the relationship between OP and support for political violence was mediated in both samples by self‐identification with the villain (Study 1A: *β* = .11, *SE* = .04, 95% CI [.04, .21]; Study 1B: *β* = .04, *SE* = .02, CI [.005, .11])—all other indirect effects through the other moral identities were not significant (all 95% CI included 0).

While Study 1 provides correlational evidence linking OP to villain identity and support for political violence, it remains unclear whether OP directly causes individuals to adopt a villainous identity. To address this gap, Study 2 employs an experimental design to manipulate passion mindsets and test the causal relationship between OP and moral identity.

## STUDY 2

Study 2 tested the causal role of passion in shaping moral identity and support for political violence. Because the victim and beneficiary roles did not consistently relate to political violence in Studies 1A and 1B, we focused here on the contrast between hero and villain identities. Whereas Studies 1A and 1B established correlational links, Study 2 experimentally manipulated passion to examine whether inducing an OP mindset would increase identification with villains and support for political violence. We hypothesized that participants primed with OP would report stronger villain identity and greater violent endorsement compared to those in a HP condition.

### Methods

#### Participants

Assuming medium effect sizes (*r* = .30) and 80% power, a sample size of 229 participants was recommended based on 5000 Monte Carlo simulations. Participants were recruited for this study after self‐identifying as supporters of the environmental cause in an MTurk pre‐screening survey. Data quality was ensured using CloudResearch's platform (Litman et al., 2017), which applied filters such as high approval ratings and exclusion of duplicate IP addresses. A total of 315 participants completed the survey (161 women, 150 men, 3 other, 1 missing; *M*_age_ = 42.39 years, *SD*_age_ = 12.04).

#### Procedure and materials

Participants were invited to take part in a study on political attitudes and were randomly assigned to one of two experimental conditions, both of which have been shown in prior research to activate different passion mindsets (Bélanger et al., 2013a, 2013b; Lafrenière et al., 2012; Schellenberg et al., 2016).

In the HP condition, the participants (*N* = 166) were instructed to follow these instructions:

Write about a time when your involvement in the environmental cause was in harmony with other things that are part of you and you felt that your involvement in the environmental cause allowed you to live a variety of experiences. Recall this time vividly and include as many details as you can to relive the experience. If this had never happened to you, imagine what such an event would feel like.

In the OP condition (*N* = 149), these instructions were as follows.

Write about a time when you had difficulties controlling your urge to get involved in the environmental cause and you felt that your involvement in the environmental cause was the only thing that really captivated you. Recall this time vividly and include as many details as you can to relive the experience. If this had never happened to you, imagine what such an event would feel like.

After the recall task, moral identity was assessed with vignettes depicting characters in heroic or villainous roles. We initially included four images from the MIPS, but one vignette showed weak internal consistency with the others. To improve reliability, we dropped that vignette and retained three images, yielding coherent scales for hero identity (α = .75) and villain identity (α = .68). Participants then completed the support for political violence scale (α = .84).

### Results and discussion

The hypothesized model was tested using AMOS (Arbuckle, 2007), with all predictor variables standardized. Table 2 presents the means, standard deviations and correlations for all measures. The model demonstrated a good fit to the data (χ^2^(1) = .54, *p* = .46, RMSEA = .00, SRMR = .01, CFI = 1.00, TLI = 1.00).

**TABLE 2:** Means, standard deviations and correlations involving variables in Study 2 (*N* = 315).

	*M*	*SD*	2	3	4
Experimental condition^a^ (1)	–	–	−.04	.14**	.08
Hero (2)	3.18 (3.11)	.64 (.69)		−.43***	−.14**
Villain (3)	1.22 (1.35)	.37 (.51)			.33***
Support for Political Violence (4)	1.40 (1.56)	.89 (.93)			

^a^ 0 = harmonious passion; 1 = obsessive passion. Means and standard deviations for participants in the harmonious and obsessive passion conditions appear outside and inside parentheses, respectively.

*p < .05, **p < .01, ***p < .001.

**FIGURE 2:**
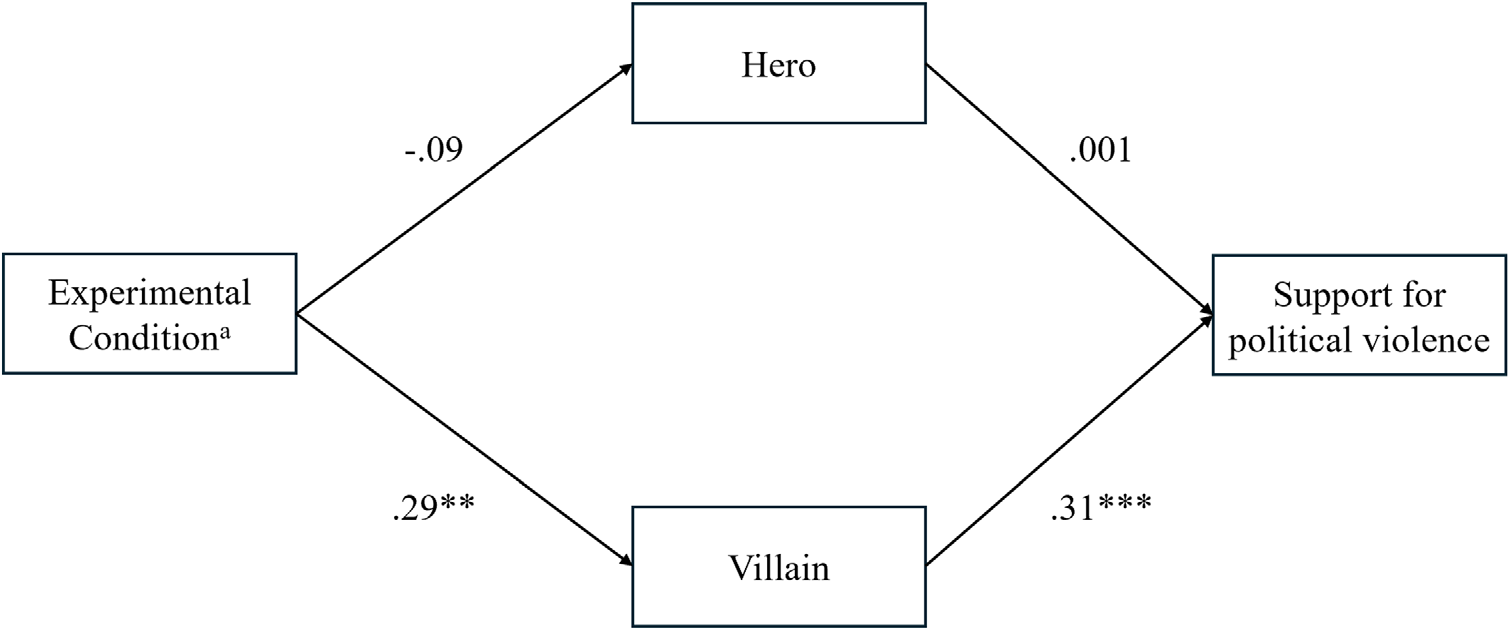
Path analysis between experimental condition and support for political violence mediated by villain identification (Study 2). ^A^0 = harmonious passion condition; 1 = obsessive passion condition, **p < .01, ***p < .001. Standardized betas are presented. Covariates are not shown in the figures for clarity.

As shown in Figure 2, results indicated that there were no differences in terms of self‐identification with the hero in the OP condition (*M* = ‐.05, *SD* = 1.03) compared to the HP condition (*M* = .04, *SD* = .96), *β* = −.09, *p* = .38. However, the results indicated that people in the OP condition (*M* = .15, *SD* = 1.13) reported a greater identification with the villain than in the HP condition (*M* = ‐.13, *SD* = .83), *β* = .29, *p* = .008. Self‐identification with the hero was not related to support for political violence (*β* = .001, *p* = .98), but identification with the villain was related to it (*β* = .31, *p* < .001).

Bias‐corrected 95% bootstrap confidence interval estimates were then calculated to test the significance of indirect effects (Preacher & Hayes, 2008). The results indicated that the relationship between OP and support for political violence was mediated by self‐identification with the villain (*β* = .09, *SE* = .04, 95% CI [.02, .19]).

Although Study 2 confirms that OP can lead to villainous self‐identification, it does not directly address whether adopting a villain identity causes greater support for political violence. To explore this causal pathway further, Study 3 experimentally manipulates moral identity itself to assess the impact of a villain mindset on participants' support for violent actions.

## STUDY 3

Study 3 tested the causal role of moral identity in shaping support for political violence using a double‐randomization design (Bullock et al., 2010; Pirlott & MacKinnon, 2016). Whereas Study 2 manipulated passion, here we manipulated identity directly by randomly assigning participants to adopt either a villain or a hero role and then measuring support for violence. This design allowed us to strengthen causal inference regarding the mediational chain from passion to identity to support for political violence by manipulating both the independent variable and the mediator across studies. We hypothesized that adopting a villain identity would increase support for political violence, reflecting the idea that villainous self‐concepts normalize violence when reframed as justified. By contrast, adopting a hero identity was expected to decrease or leave support for violence unchanged, consistent with the notion that heroic roles preserve conventional moral boundaries.

### Methods

#### Participants

Assuming medium effect sizes (*d* = .50) and 80% power, a sample size of 102 participants was recommended using G*Power (Faul et al., 2009). Participants were recruited for this study after self‐identifying as supporters of the Black Lives Matter movement in an MTurk pre‐screening survey. Data quality was ensured using CloudResearch's platform (Litman et al., 2017), which applied filters such as high approval ratings and exclusion of duplicate IP addresses. A total of 179 participants completed the survey (97 women, 78 men, 4 others; *M*_age_ = 38.96 years, *SD*_age_ = 11.85).

#### Procedure and materials

Participants were invited to take part in a study on political attitudes and were randomly assigned to one of two experimental conditions to activate different moral identities (Gray, 2010).

In the villain condition (*N* = 85), participants were instructed to follow these instructions:

Please write a brief fictional story with you as the lead character. In this story, you should describe a scene in which you harm another person. Try to write the story so that whoever reads it gets the impression that you are truly evil. The story should be realistic.

In the control condition (*N* = 94), these instructions were:

Please write a brief fictional story with you as the lead character. In this story, you should describe a scene in which you have lunch with another person. Try to write the story so that whoever reads it gets the impression of the lunch. The story should be realistic.

After the recall task, participants completed the support for political violence scale (α = .79). As a manipulation check, participants were shown four pictures from the Moral Identity Picture Scale, each including two characters, a hero and a villain. For each picture, participants indicated to what extent they identified with the hero (α = .73) and the villain (α = .76).

### Results and discussion

We tested our predictions using a MANOVA, which examined the effect of the experimental conditions on the manipulation check and support for political violence. Table 3 presents the means, standard deviations and correlations for all measures.

**TABLE 3:** Means, standard deviations and correlations involving variables in Study 3 (*N* = 179).

	*M*	*SD*	2	3	4
Experimental condition^a^ (1)	–	–	.10	.18*	.17*
Hero (2)	2.86 (3.01)	.76 (.61)		−.22**	.11
Villain (3)	1.21 (1.38)	.42 (.51)			.39***
Support for Political Violence (4)	1.77 (2.19)	1.00 (1.34)			

^a^ 0 = control condition; 1 = villain condition. Means and standard deviations for participants in the control and villain conditions appear outside and inside parentheses, respectively.

*p < .05, **p < .01, ***p < .001.

Regarding the manipulation check, the results indicated that participants in the villain condition (*M* = 1.38, *SD* = .51) identified more with villains than participants in the control condition (*M* = 1.21, *SD* = .42), *F*(1, 177) = 6.04, *p* = .01, partial eta‐square = .03. As expected, the experimental manipulation did not influence the extent to which participants identified themselves with the heroes, *F*(1, 177) = 2.01, *p* = .15.

Regarding the effect of the experimental manipulation on support for political violence, results revealed that participants in the villain condition (*M* = 2.19, *SD* = 1.34) reported greater support for political violence than participants in the control condition (*M* = 1.77, *SD* = 1.00), *F*(1, 177) = 5.74, *p* = .01, partial eta‐square = .03.

While Study 3 demonstrates that adopting a villain identity increases support for political violence, it does not explain why individuals with OP are more inclined to identify with villains in the first place. To address this question, Study 4 examines the underlying psychological mechanisms—specifically, how OP affects perceptions of villains' warmth, competence and status, which in turn fosters villain identification.

## STUDY 4

The goal of Study 4 was to examine why individuals with ideological obsession are more likely to identify with a villain (vs. hero) moral identity. To investigate this, we used Fiske's Stereotype Content Model (SCM) (Fiske et al., 2002), which posits that people assess others along three core dimensions: competence, warmth and status. We predicted that obsessive (but not harmonious) passion would be related to stronger perceptions of competence, warmth and status in villains, leading to greater villain identification, which in turn would increase support for political violence.

We base these predictions on previous research that has shown that OP is strongly linked to basic psychological needs frustration, particularly deficits in competence, autonomy and relatedness (Lalande et al., 2017). Individuals experiencing OP often derive their self‐worth from their ideological pursuits, which makes them vulnerable to feelings of inadequacy when those needs are unmet. These individuals may experience a lack of control over their lives (low autonomy), feel disconnected from others (low relatedness) or perceive themselves as ineffective or powerless (low competence). To compensate for these frustrations, they may gravitate towards villainous characters, who are perceived as embodying competence, agency and prestige (Fiske et al., 2007). Villains are often depicted as decisive and powerful figures who assert control over their environment, which can appeal to individuals struggling with a sense of personal inefficacy.

Furthermore, villains are not only seen as competent but also as righteous in their own moral framework. While traditionally viewed as agents of moral transgression, villains often act with moral conviction, believing that their extreme actions—such as punishing wrongdoers—are justified and necessary for achieving the greater good. This moral certainty and willingness to cross ethical boundaries can resonate with individuals driven by OP, who may similarly view their ideological cause as warranting uncompromising, even violent actions. The alignment between the villain's sense of purpose and the obsessive individual's need to affirm their ideological significance could make the villain's identity particularly attractive. Thus, it may provide them with a sense of empowerment and moral clarity, despite the transgressive nature of their actions, providing a psychological pathway towards support for political violence.

Given these arguments, we made two key predictions. First, we hypothesized that OP would be related to perceiving greater competence, warmth and status in the villain (vs. hero), which would be associated with identification with the villain. Second, we predicted that this identification with the villain would, in turn, be related to support for political violence. These hypotheses extend the findings from Studies 1–3, offering a clearer understanding of the pathways linking OP, villain identification and the endorsement of support for political violence.

### Method

#### Participants

As in previous studies, we expected large correlations (*r* = .50) between the independent predictors, OP and Villains, medium effect sizes effects (*r* = .30) were hypothesized between warmth, competence, status and violent extremism; 185 people were suggested by 5000 Monte Carlo simulations. Participants were 277 American Muslims (100 women, 176 men, 1 other; *M*_age_ = 34.61 years, *SD*_age_ = 9.03) recruited through CloudResearch's platform (Litman et al., 2017).

#### Procedure and materials

First, participants completed the obsessive (α = .91) and harmonious (α = .92) passion Scales, along with the ideological commitment scale (α = .86). They were then presented with a vignette from the Moral Identity Picture Scale depicting a hero and a villain. Because the survey already included several additional measures (competence, warmth and status), we limited the moral identity assessment to a single vignette from the Moral Identity Picture Scale depicting a hero and a villain in order to avoid participant fatigue. Using the scales developed by Fiske et al. (2002), participants rated the hero and villain on competence (e.g. competent, confident, independent, competitive, intelligent), warmth (e.g. tolerant, warm, good‐natured, sincere) and status (e.g. works in a prestigious job, economically successful, well‐educated) on a scale ranging from 1 (*strongly disagree*) and 5 (*strongly agree*). The scales demonstrated reliability for both the hero (competence: α = .80, warmth: α = .89, status: α = .83) and the villain (competence: α = .83, warmth: α = .86, status: α = .89). Participants then indicated the extent to which they identify with the hero and the villain and completed the support for political violence scale (α = .94).

### Results and discussion

AMOS was used to test the proposed model (Arbuckle, 2007), with all predictor variables standardized. The means, standard deviations and correlations for all measures are presented in Table 4.

**TABLE 4:** Means, standard deviations and correlations involving variables in Study 4 (*N* = 277) and Study 5 (pre‐registered; *N* = 294).

	*M*	*SD*	2	3	4	5	6	7	8	9	10	11	12
Obsessive Passion (1)	3.28 (1.96)	1.64 (1.21)	.39*** (.51***)	.51*** (.62***)	−.07 (−.16**)	.27*** (.07)	.13* (−.08)	.19*** (.15**)	−.00 (−.04)	.32*** (.20***)	.27*** (.17**)	.13* (.05)	.47*** (.35***)
Harmonious Passion (2)	5.21 (4.06)	1.23 (1.46)		.79*** (.79***)	.03 (−.00)	−.06 (.00)	−.01 (−.03)	.29*** (.14*)	.36*** (.12*)	.20*** (.15**)	−.01 (.10)	.17** (.09)	.07 (.18***)
Commitment (3)	5.21 (3.80)	1.31 (1.32)			−.01 (−.07)	−.02 (−.03)	−.02 (−.10)	.31*** (.18**)	.32*** (.12*)	.26*** (.19***)	.00 (.11)	.19*** (.09)	.17** (.25***)
Villain Competence (4)	2.94 (3.23)	1.01 (.93)				.46*** (.42***)	.57*** (.63***)	−.24*** (−.28***)	−.13* (−.03)	−.34*** (−.20***)	.29*** (.20***)	−.20*** (−.04)	.13* (−.08)
Villain Warmth (5)	1.71 (1.89)	.92 (.74)					.62*** (.38***)	−.35*** (−.25***)	−.50*** (−.43***)	−.23*** (−.13*)	.60*** (.33***)	−.24*** (−.31***)	.53*** (.12*)
Villain Status (6)	2.21 (2.93)	1.03 (.90)						−.26*** (−.19***)	−.28*** (−.07)	−.30*** (−.22***)	.42*** (.21***)	−.18** (−.09)	.30*** (−.11*)
Hero Competence (7)	3.89 (3.74)	.72 (.68)							.66*** (.55***)	.66*** (.58***)	−.18** (−.26***)	.38*** (.43***)	−.09 (.04)
Hero Warmth (8)	4.28 (4.38)	.74 (.64)								.46*** (.42***)	−.23*** (−.23***)	.39*** (.43***)	−.24*** (−.06)
Hero Status (9)	3.61 (3.28)	.80 (.71)									−.10 (−.14*)	.41*** (.24***)	.01 (.12*)
Identification with Villain (10)	1.31 (1.33)	.70 (.62)										−.23*** (−.41***)	.49*** (.15**)
Identification with Hero (11)	3.14 (3.15)	.96 (.84)											−.05 (−.05)
Support for Political Violence (12)	1.98 (1.25)	1.64 (.80)											

*Note*: Values for Muslims (Study 4) and Republicans (Study 5) are located outside and inside the parentheses, respectively.

*p < .05, **p < .01, ***p < .001.

We conducted two complementary analyses. In the first analysis, we used a difference score between hero and villain to examine relative perceptions, revealing whether participants identified more strongly with the villain by contrasting them with heroes. This highlights the comparative appeal of villains over heroes. In the second analysis, we focused solely on the villain, examining their absolute appeal independent of hero comparisons. This allowed us to isolate specific qualities, such as warmth and status, that directly drive identification with villains and their link to political violence. Together, these approaches provide a more complete understanding of villain identification.

#### Relative identification: Villain vs. hero

In this first analysis, we examined why individuals identify with hero or villain characters. To that end, we tested a model linking ideological commitment, OP and HP to differences in perceived competence, warmth and status between the hero and villain. These difference scores were then associated with variations in character identification.

**FIGURE 3:**
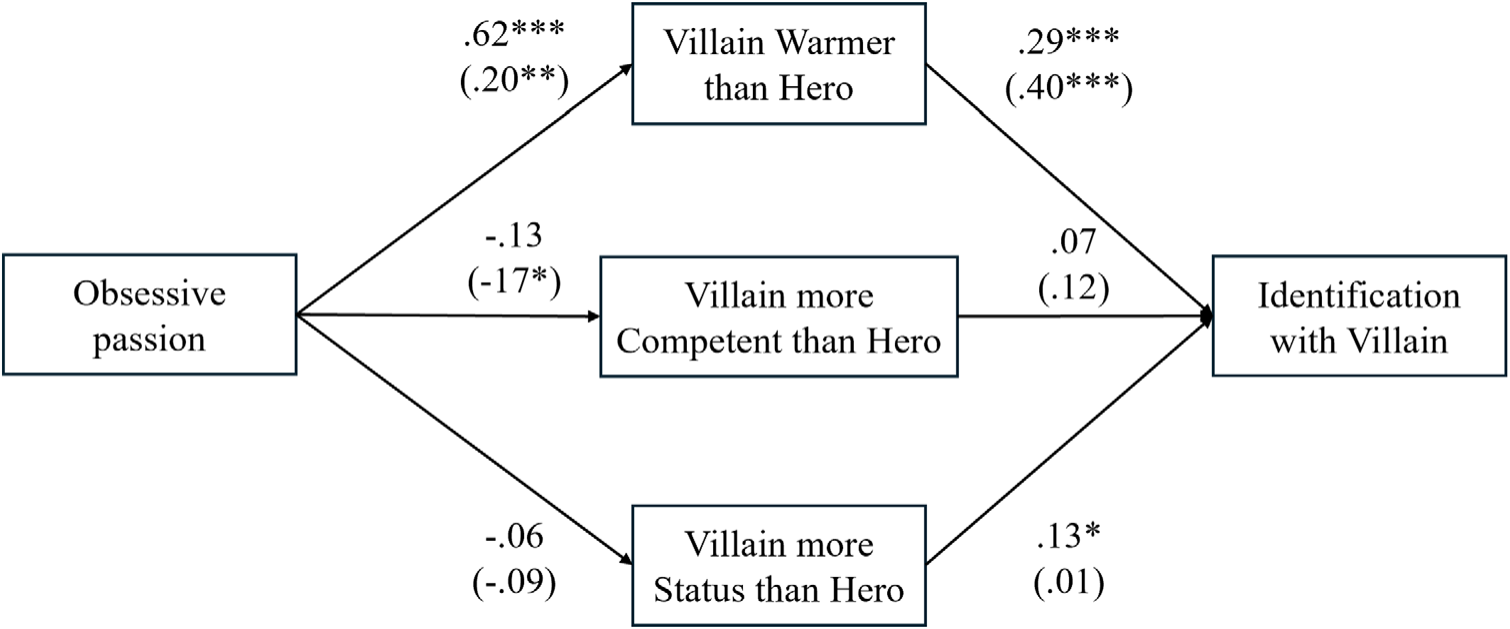
Path analysis between obsession passion, relative identification with villain and hero (Studies 4–5). *p < .05, **p < .01, ***p < .001. Standardized betas are shown. Ideological commitment and harmonious passion were included in the analyses; for clarity, only selected paths are displayed. Full models are reported in the text.

The model provided a good fit to the data, *χ* ^2^ (3) = .76, *p* = .85, RMSEA = .00, SRMR = .00, CFI = 1.00, TLI = 1.00. As shown in Figure 3, OP was positively related (*β* = .62, *p* = .001), while HP was negatively related (*β* = −.35, *p* = .02) to perceiving the villain as warmer than the hero. Ideological commitment was negatively related to perceiving the villain as warmer than the hero (*β* = −.38, *p* = .02)—all other relationships linking ideological passion and commitment to the perception of the villain and the hero were not significant (all *ps* > .11).

In terms of identification with the villain rather than the hero, the results showed that perceiving the villain as warmer than the hero was related to identification with the villain rather than the hero (*β* = .29, *p* = .001). Perceiving the villain as having more status than the hero was also positively related to identification with the villain rather than the hero (*β* = .13, *p* = .03). Perceiving the villain as more competent than the hero did not predict identification with the villain (*β* = .07, *p* = .23).

Bias‐corrected 95% bootstrap confidence interval estimates were then calculated to test the significance of the indirect effect (Preacher & Hayes, 2008). Results indicated that the relationship between OP and identification with the villain rather than the hero was mediated by perceiving the villain as warmer than the hero (*β* = .18, *SE* = .04, 95% CI [.10, .28]).

#### Absolute identification: The appeal of villains

**FIGURE 4:**
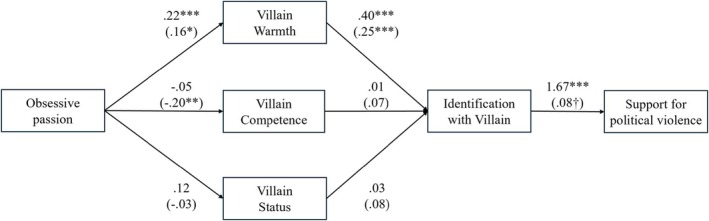
Path analysis between obsession passion, absolute identification with villain and hero and support for political violence (Studies 4–5). *p < .05, **p < .01, ***p < .001. Standardized betas are shown. Ideological commitment and harmonious passion were included in the analyses; for clarity, only selected paths are displayed. Full models are reported in the text.

In the second analysis, we tested the model, where OP is linked to perceived warmth of the villain, which in turn is associated with identification with the villain, and support for political violence. An error covariance was estimated between identification with the villain and support for political violence. The model provided a good fit to the data, *χ* ^2^ (4) = 2.42, *p* = .65, RMSEA = .00, SRMR = .01, CFI = 1.00, TLI = 1.00. As shown in Figure 4, OP was positively associated with perceiving the villain as warm (*β* = .22, *p* < .001) and having status (*β* = .12, *p* = .005), but not competence (*β* = −.05, *p* = .21). HP and ideological commitment were not related to competence, warmth or status (all *p* > .10). The perceived warmth of the villain (*β* = .40, *p* < .001) was related to identification with the villain, but not competence (*β* = .01, *p* = .69) or status (*β* = .05, *p* = .41). Identification with the villain was associated with support for political violence (*β* = 1.68, *p* < .001).

Bias‐corrected 95% bootstrap confidence interval estimates were then calculated to test the significance of the indirect effect (Preacher & Hayes, 2008). Results indicated that the mediated relationship between OP and support for political violence through perceived warmth of the villain and identification with the villain was significant (*β* = .15, *SE* = .04, 95% CI [.08, .25]).

Although Study 4 provides valuable insights into the psychological processes linking OP to villain identification, it remains uncertain whether these findings generalize across different ideological contexts. To test the robustness and reliability of these results, Study 5 was preregistered and replicates the design of Study 4 with a politically distinct sample—Republicans.

## STUDY 5

The purpose of Study 5 was twofold. First, we aimed to replicate the findings of Study 4, which focused on a religious group (i.e. Muslims), by testing a political group (i.e. Republicans) to assess the generalizability of our results across different ideological contexts. Second, to ensure that the results from Study 4 were not influenced by the specific moral identity imagery used, we employed a different visual stimulus—still featuring a hero and a villain, but with the villain now placed on the right and the hero on the left (the reverse of Study 4). This adjustment was made to eliminate potential presentation biases. As in Study 4, we used only one vignette in order to keep the survey length manageable, given the inclusion of additional measures of competence, warmth and status. Additionally, we preregistered our hypotheses and analytical plan on AsPredicted.org to enhance transparency and methodological rigour: https://aspredicted.org/m3f8‐ywd3.pdf.

### Methods

#### Participants

In this study, we preregistered to recruit 285 participants, which was 100 more than the number suggested by the power analysis conducted in Study 4. A total of 294 participants were recruited, as 9 individuals completed the study before data collection was closed (total *N* = 294; 161 women, 133 men; *M*_age_ = 44.55 years, *SD*_age_ = 13.88). Participants were recruited through the CloudResearch platform (Litman et al., 2017) and had self‐identified as Republicans in a prescreening survey.

#### Procedure and materials

First, participants completed the OP scale (α = .89), HP scale (α = .92) and the ideological commitment scale (α = .84). They were then presented with a vignette from the Moral Identity Picture Scale depicting a hero and a villain. Using scales developed by Fiske et al. (2002), participants rated the hero and villain on competence (e.g. competent, confident, independent, competitive, intelligent), warmth (e.g. tolerant, warm, good‐natured, sincere) and status (e.g. works in a prestigious job, economically successful, well‐educated). The scales showed good reliability for both the hero (competence: α = .80, warmth: α = .84, status: α = .85) and the villain (competence: α = .86, warmth: α = .79, status: α = .92). As in Study 4, participants then indicated the extent to which they identified with the hero and villain. Finally, they completed the support for political violence scale (α = .92).

### Results and discussion

AMOS was used to test the proposed model (Arbuckle, 2007), with all predictor variables standardized. The means, standard deviations and correlations for all measures are presented in Table 4. Akin to Study 4, we conducted two complementary analyses to better understand villain identification. First, we used a difference score between hero and villain to examine relative perceptions and second, we focused solely on the villain's absolute appeal.

#### Relative identification: Villain vs. hero

As in Study 4, in this first analysis, we examined why individuals identify with hero or villain characters. We tested a model linking ideological commitment, OP and HP to differences in perceived competence, warmth and status between the hero and villain, with these differences then associated with variations in character identification.

The model provided a good fit to the data, *χ* ^2^(3) = 1.58, *p* = .66, RMSEA = .00, SRMR = .01, CFI = 1.00, TLI = 1.00. As shown in Figure 3, OP was positively related (*β* = .20, *p* = .005), while commitment was negatively related (*β* = −.21, *p* = .03), to perceiving the villain as warmer than the hero. OP was negatively related to perceiving the villain as more competent than the hero (*β* = −.17, *p* = .01) and ideological commitment was negatively and marginally related to perceiving the villain as having more status than the hero (*β* = −.19, *p* = .06) —all other relationships linking passion and ideological commitment to the perception of the villain and the hero were not significant (all *ps* > .19).

In terms of identification with the villain rather than the hero, the results showed that perceiving the villain as warmer than the hero was related to identification with the villain rather than the hero (*β* = .40, *p* < .001). Perceiving the villain as having more status was not related to identification with the villain rather than the hero (*β* = .01, *p* = .79); perceiving greater competence in the villain rather than the hero was marginally related to identifying more with the villain rather than the hero (*β* = .12, *p* = .06).

Bias‐corrected 95% bootstrap confidence interval estimates were then calculated to test the significance of the indirect effect (Preacher & Hayes, 2008). Results indicated that the relationship between OP and identification with the villain rather than the hero was mediated by perceiving the villain as warmer than the hero (*β* = .08, *SE* = .04, 95% CI [.01, .17]).

#### Absolute identification: The appeal of villains

Like the previous study, in the second analysis, we tested a model where OP is linked to the perceived warmth of the villain, which in turn is associated with villain identification and support for political violence. An error covariance was estimated between status and support for political violence. The model provided a good fit to the data, *χ* ^2^ (4) = 4.76, *p* = .31, RMSEA = .02, SRMR = .01, CFI = .99, TLI = .99. As shown in Figure 4, OP was positively associated with perceiving the villain as warm (*β* = .16, *p* = .02), negatively related to being competent (*β* = −20, *p* = .005) and not related to perceived status (*β* = −.03, *p* = .63). HP was marginally associated with the perception of the villain as competent (*β* = .16, *p* = .07) and ideological commitment was marginally and negatively associated with the perception of the villain as warm (*β* = −.19, *p* = .06)—all other *p*s > .09. Perceived warmth of the villain was related to identification with the villain (*β* = .25, *p* < .001), but competence (*β* = .07, *p* = .30) and status (*β* = .08, *p* = .22) were not. Identification with the villain was marginally associated with supporting political violence (*β* = .08, *p =* .07).

Bias‐corrected 95% bootstrap confidence interval estimates were then calculated to test the significance of the indirect effect (Preacher & Hayes, 2008). Results indicated that the mediated relationship between OP and support for political violence through perceived warmth of the villain and identification with the villain was significant (*β* = .003, *SE* = .004, 95% CI [.0001, .016]).

## STUDY 6

Study 6 tested whether villain identity reflects a coherent moral orientation through systematic patterns of moral inversion. Whereas earlier studies established correlational and experimental links between OP, moral identity and support for political violence, this study examined whether villain and hero identities are tied to consistent patterns of moral appraisal that, in turn, predict violent endorsement.

We hypothesized that OP would predict greater villain identity, which would be associated with moral inversion: villains recast as justified and not harmful and heroes recast as harmful or misguided. These reappraisals were expected to predict greater support for political violence, thereby situating villain identity within a broader process by which passion translates into violent endorsement. In contrast, HP was expected to predict greater hero identity, which would correspond to more conventional moral appraisals—heroes seen as justified and not harmful and villains seen as harmful—thereby preserving traditional moral boundaries.

### Methods

#### Participants

As in prior studies, we anticipated large correlations (*r* = .50) between OP and villain identity and medium effect sizes (*r* = .30) for associations between perceived harm and justification. Monte Carlo simulations (5000 iterations) indicated that a sample of 185 participants would provide adequate power. Participants were recruited through MTurk after self‐identifying as supporters of the environmental cause in a pre‐screening survey, with data quality ensured via CloudResearch's platform (Litman et al., 2017). The final sample included 236 participants (112 women, 123 men, 1 other; *M*_age_ = 42.77, *SD* = 11.27).

#### Procedure and materials

##### Passion

As in previous studies, the passion scale (Vallerand et al., 2003) was used to measure harmonious (α = .86) and OP (α = .93).

##### Commitment

As in previous studies, participants reported the degree to which they are committed to their ideology (α = .80).

##### Moral identity

Participants were shown the same four vignettes used in Studies 2–3, each depicting a hero and a villain. After viewing each image, they first indicated how much they identified with each character (hero α = .69, villain α = .88) on a 4‐point scale ranging from 1 (*not at all*) to 4 (*extremely*). They then evaluated each character's actions on two dimensions: harm and justification. The harm measure included three items (*causing harm to others*, *having negative consequences for innocent people*, *being aggressive and violent*). The justification measure also included three items (*justified given the circumstances*, *necessary to achieve a greater good*, *unavoidable to achieve an important objective*). All items were rated on a 4‐point scale (1 = *not at all*, 4 = *extremely*). Items were averaged separately for heroes and villains, yielding four composites: Villain Harm (α = .84), Hero Harm (α = .94), Villain Justified (α = .93) and Hero Justified (α = .87).

##### Support for political violence

As in previous studies, participants' support for political violence was measured using five items (α = .96).

### Results and discussion

AMOS was used to test the proposed model (Arbuckle, 2007), with all predictor variables standardized. The means, standard deviations and correlations for all measures are presented in Table 5. In this model, obsessive and harmonious passion predicted villain and hero identity, which in turn were specified to predict role‐specific moral appraisals (Villain Harm, Villain Justified, Hero Harm, Hero Justified). These appraisals, together with identities, were modelled as predictors of support for political violence. The hypothesized model showed acceptable fit to the data, χ^2^(9) = 21.52, *p* = .011, RMSEA = .07, SRMR = .02, CFI = .99, TLI = .96.

**TABLE 5:** Means, standard deviations and correlations involving variables in Study 6 (*N* = 236).

	*M*	*SD*	2	3	4	5	6	7	8	9	10
Obsessive Passion (1)	3.25	1.75	.42***	.52***	−.20**	.68***	.71***	−.05	.70***	.15*	.77***
Harmonious Passion (2)	4.98	1.09		.80***	.12	.17**	.09	.22***	.18**	.32***	.32***
Commitment (3)	4.89	1.19			.05	.25***	.21***	.14*	.31***	.24***	.41***
Harm Villain (4)	3.30	.52				−.47***	−.32***	.37***	−.29***	.35***	−.32**
Harm Hero (5)	1.62	.78					.72***	−.04	.72***	.02	.76***
Justified Villain (6)	1.91	.81						−.25***	.79***	−.07	.75***
Justified Hero (7)	2.89	.63							−.10	.43***	−.04
Identification with Villain (8)	1.82	.95								−.09	.81***
Identification with Hero (9)	3.00	.69									.07
Support for Political Violence (10)	2.54	2.03									

*p < .05, **p < .01, ***p < .001.

**FIGURE 5:**
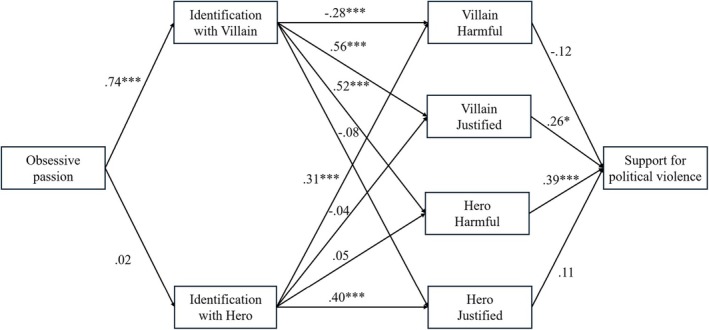
Path model of obsessive and harmonious passion, moral identity, moral appraisals and support for political violence (Study 6). *p < .05, ***p < .001. Standardized betas are shown. Ideological commitment and harmonious passion were included in the analyses; for clarity, only selected paths are displayed. Full models are reported in the text.

As shown in Figure 5, OP positively predicted villain identity (β = .74, *p* < .001). HP negatively predicted villain identity (β = −.19, *p* = .01) and positively predicted hero identity (β = .35, *p* < .001). Villain identity predicted lower villain harmfulness (β = −.28, *p* < .001), higher villain justification (β = .57, *p* < .001) and higher hero harmfulness (β = .52, *p* < .001), but was not significantly related to hero justification (β = −.08, *p* = .18). Hero identity predicted higher hero justification (β = .40, *p* < .001) and higher villain harmfulness (β = .31, *p* < .001), but was unrelated to hero harmfulness (β = .05, *p* = .22) or villain justification (β = −.04, *p* = .27).

Support for political violence was significantly predicted by villain justification (β = .26, *p* = .03) and hero harmfulness (β = .39, *p* < .001), whereas villain harmfulness (β = −.12, *p* = .11) and hero justification (β = .11, *p* = .12) were not significant. Villain identity also exerted a direct positive effect on support for political violence (β = .72, *p* < .001), as did OP (β = .49, *p* < .001).

Bootstrapped mediation analyses (5000 samples) revealed two significant indirect effects of OP on support for political violence. First, OP predicted greater villain identity, which in turn predicted greater villain justification, which was associated with support for political violence (β = .11, SE = .06, 95% CI [.01, .25]). Second, OP predicted greater villain identity, which in turn predicted greater hero harmfulness, which was associated with support for political violence (β = .15, SE = .05, 95% CI [.06, .27]).

Taken together, these findings provide support for the moral inversion hypothesis. Individuals high in OP were more likely to adopt a villainous identity, which was systematically tied to recasting villains as justified and not harmful and heroes as harmful. Crucially, these moral reappraisals were not incidental—they formed the psychological bridge from identity to the endorsement of political violence. The indirect effects showed that OP predicted greater support for political violence because villain identity reshaped moral meaning: villains were seen as justified and heroes as harmful, which in turn made violence appear warranted. In other words, it was not simply adopting a villainous identity that mattered, but the way this identity redefined morality so that violence could be endorsed as legitimate. In contrast, HP predicted hero identity, which was associated with more conventional appraisals—heroes were judged as justified, and villains were judged as harmful. This pattern suggests that HP preserves traditional moral categories and therefore protects against violent endorsement, whereas OP fosters a reinterpretation of morality that normalizes violence.

## GENERAL DISCUSSION

This research provides important new perspectives on the link between ideological passion and moral identity, focusing on how OP can foster support for political violence. Across six studies, we demonstrated that OP, but not HP, was consistently related to the adoption of a villainous moral identity and that this identity was systematically tied to moral reappraisals that made violence appear warranted. While individuals often strive to view themselves as ‘heroes’, embodying justice, empathy and moral righteousness because it aligns with socially valued traits and fosters a positive moral self‐concept (Hitlin, 2007), those with OP were more likely to adopt a villainous self‐concept—one where violence was redefined as a justified means to achieve ideological goals. This shift from a conventional moral identity to a transgressive one reflects a form of moral inversion, whereby villains are construed as justified and not harmful and heroes are reframed as harmful and misguided. In this sense, the pathway to violent extremism does not only rest on disengagement from moral standards but on the active redefinition of moral categories themselves.

The six studies converged on this conclusion through complementary methods. Studies 1–3 established the basic and causal link between OP, villain identity and support for political violence. Studies 4 and 5 showed why villains become appealing to OP individuals, identifying perceptions of warmth as a key factor and replicated these findings in a preregistered design. Finally, Study 6 provided the most stringent test of construct validity by showing that villain identity was not a superficial labelling artefact of the Moral Identity Picture Scale but was embedded in a coherent pattern of moral appraisals. Importantly, this interpretation aligns with prior qualitative evidence that perpetrators of violence rarely see themselves as immoral but as justified transgressors acting under a moral mandate (Baumeister, 1997; Monroe, 2008; Rai et al., 2017). Crucially, these reappraisals served as the psychological bridge from identity to the endorsement of violence: villains were seen as justified, heroes were seen as harmful and these judgements predicted support for political violence. In contrast, HP predicted hero identity, which was associated with more conventional appraisals—heroes judged as justified and villains as harmful—thereby preserving traditional moral categories and offering protection against violent endorsement. These findings advance understanding of how passion and moral identity interact to shape violent endorsement and they carry important implications for both theory and intervention.

### Theoretical contribution

The first contribution of this research is to refine how we understand moral self‐identification among ideologically committed individuals. Our findings show that individuals with OP identify with roles that society conventionally associates with villains. Importantly, this does not mean that they embrace themselves as evil or harmful. Rather, OP is associated with a process of moral inversion in which villainous roles are reinterpreted as justified, while heroic roles can be recast as harmful or misguided. Villain identity in this sense represents not an abandonment of morality but a redefinition of its boundaries: sustaining a moral self‐concept through the appropriation of conventionally transgressive roles. By contrast, HP supports conventional hero identities, in which heroes are viewed as good and villains as bad.

This supports theories of violent extremism, suggesting that moral self‐concept is not lost in radicalization but transformed. To do violence for one's cause, individuals must reconfigure their moral framework—exchanging mainstream morality, which condemns violence, for the group's moral code, which justifies it as necessary and good (see Moghaddam, 2005's ‘Third Floor’). OP individuals are especially prone to this shift because they are drawn to violence‐justifying groups that normalize and reinforce moral systems legitimizing violence (Bélanger et al., 2020). Within such groups, members adopt roles society labels ‘villainous’ but experience them as heroic—acts of moral duty in service of a higher cause. Consistent with this, OP individuals are more likely to endorse violent strategies on utilitarian rather than deontological grounds (Snook et al., 2025). When they identify as villains, they are not embracing evil but engaging in instrumental moral reasoning—choosing what they see as necessary means to achieve a greater good.

The findings from Study 4 and Study 5 help explain why individuals driven by OP tend to identify with the villain. These studies reveal that individuals with OP are more likely to perceive villains as possessing warmth, a trait traditionally associated with positive moral agents, and measured by characteristics such as tolerance, sincerity, being good‐natured and being sincere. Rather than viewing villains as cold, detached or purely evil, ideologically obsessed individuals see them as relatable and sincere figures who act with a sense of righteousness in pursuit of their goals. This attribution of warmth to the villain challenges traditional stereotypes and helps explain why these individuals align themselves with a villainous identity. The more sympathetic (rather than competent or high‐status) one finds villains, the more alluring the role becomes. The perception of warmth makes the villain a more attractive moral role model, allowing ideologically obsessed individuals to justify their transgressive actions within a moral framework that includes sincerity and righteousness.

The second contribution of this research is to propose a new psychological mechanism that links OP to support for political violence. Prior work has shown that OP predicts violent endorsement through moral disengagement (Bélanger, Schumpe, & Nisa, 2019; Bélanger, Schumpe, Nociti, et al., 2019), whereby individuals detach from conventional moral standards to justify harm. Our findings suggest a complementary process: OP can also foster villain identity, in which individuals do not merely loosen moral constraints but actively redefine the moral meaning of villainy itself. This indicates that support for violence may emerge not only from a weakening of moral norms but also from a reconfiguration of moral categories.

This mechanism, grounded in moral inversion, involves reshaping moral boundaries so that villainous roles are recast as justified and heroes as harmful. OP therefore does not simply entrench ideological rigidity—it actively transforms how individuals perceive the morality of violence. In this context, support for political violence becomes the culmination of a shift towards viewing harm as not only acceptable but necessary. By highlighting this possibility, our research enriches current models of radicalization by underscoring the moral dimensions of OP's connection to ideological violence. Moreover, this perspective helps explain why OP is consistently linked to personality traits such as narcissism and Machiavellianism, often associated with radicalism (Chabrol et al., 2020; Pavlović & Storm, 2020). Increased villainous self‐identification among high‐OP individuals suggests a deeper psychological alignment with these traits, consistent with observed connections between OP and the Dark Tetrad (Bélanger et al., 2023).

A third contribution concerns the mutability of moral identity. Traditionally, moral identity has been viewed as relatively stable and grounded in enduring values (Aquino & Reed, 2002). Our findings suggest, however, that moral identity may shift depending on the type of passion that structures ideological commitment. Under an OP mindset, individuals were more likely to move towards a villainous identity, accompanied by moral reappraisals that recast violence as justified. Under HP, individuals sustained a conventional heroic identity that resisted such reinterpretation. This pattern indicates that moral identity is more dynamic than previously assumed, with the potential to adapt to motivational pressures. Such flexibility carries important implications for theories of radicalization, which often assume fixed self‐concepts and for interventions, which may need to address how moral boundaries can be reshaped rather than simply eroded.

### Practical applications and future research

One application of this research lies in enhancing behavioural threat assessment and counter‐radicalization strategies within security and intelligence domains. Traditional risk assessment models primarily emphasize ideological content, social networks and behavioural indicators, but they often neglect the moral self‐concepts that render violence psychologically meaningful, which may explain why such models have low predictive validity (Cherney & Belton, 2024). The present findings indicate that individuals driven by OP may adopt villainous identities that redefine harm as morally necessary, signalling a distinctive moral pathway towards violent engagement. Integrating measures of moral identity and moral inversion into existing threat assessment frameworks could therefore improve the early identification of individuals at risk of ideological violence. Security analysts and counter‐extremism practitioners might be trained to recognize linguistic and narrative markers of villain identity—such as the moral justification of harm, references to ‘necessary evil’ or depictions of violence as redemptive—within interviews, digital communications or manifestos. Incorporating these indicators alongside conventional behavioural metrics could enhance predictive validity and inform more targeted deradicalization or rehabilitation interventions. By integrating moral identity into threat assessment, this approach connects psychological theory with security practice and clarifies how ideological passion and moral reasoning combine to drive violent extremism.

Beyond practical applications, this line of research opens several promising directions for future work. One is to investigate the psychological functions of villain identity. While our findings show that OP can foster villainous self‐concepts, it remains unclear what these identities provide for individuals: do they serve as sources of empowerment, distinctiveness or moral coherence in the face of ideological conflict? Understanding these functions could shed light on why some individuals willingly embrace villainous roles despite their social stigma.

A second avenue is to examine how villain and hero identities operate in group and cultural contexts. Our studies focused on individual‐level processes, but future research could also test whether groups promote villain identities through collective narratives. For example, radical environmental or anarchist movements are often portrayed as villains by authorities and future work could examine whether such external labels are sometimes embraced within the movement as markers of loyalty to a larger cause. In this way, villain identity may function not only as an individual self‐concept but also as a collective badge of belonging, suggesting that passion‐driven villain identities could be reinforced when embedded in group‐level identity projects.

Future research should also consider the dynamic trajectories of moral identity. Radicalization may not be a simple shift from hero to villain, but rather a back‐and‐forth process in which individuals alternate between seeing themselves as defenders and as necessary transgressors. Longitudinal studies could capture these oscillations, clarifying how passion interacts with changing contexts to produce shifts in self‐concept.

### Limitations

Despite these contributions, several limitations should be acknowledged. First, our reliance on self‐report measures for both moral identity and support for violence raises the possibility of social desirability bias. Participants may have underreported extremist attitudes or overstated their moral self‐perceptions, even in anonymous survey contexts. Future research could address this limitation by controlling for scores on a social desirability scale, incorporating implicit measures of moral identity or by using behavioural indicators of violent endorsement in real‐world or laboratory settings.

Second, although we examined diverse ideological groups within the United States—including Democrats, Republicans, environmental activists, Muslims and Black Lives Matter supporters—the generalizability of our findings to non‐Western contexts is uncertain. Ideological passion and moral identity may manifest differently in cultures with distinct moral foundations, such as those emphasizing collectivist values or alternative conceptions of moral agency. Future research should test the universality of our findings by investigating ideological movements in non‐Western cultural settings.

A further limitation concerns the lack of a manipulation check in Study 2. Although we relied on an established procedure that has been used successfully in prior research to induce obsessive and HP, we did not include a direct measure to confirm that the manipulation worked as intended in our sample. This means the effectiveness of the manipulation in the present context remains uncertain. Future work should incorporate manipulation checks to provide stronger evidence that the intended psychological states were successfully elicited.

Finally, while our research focused on moral identity as a mediator between passion and violent endorsement, other psychological processes—such as group identity, collective narcissism and broader intergroup dynamics—are also likely to play significant roles in radicalization. These factors were beyond the scope of our design but remain crucial for understanding the wider social and psychological environment in which passion and moral identity operate. Integrating these processes in future research would provide a more comprehensive account of the pathways to ideological violence.

## CONCLUSION

This research highlights the crucial roles of OP and moral identity in shaping support for violent extremism. Across six studies, OP consistently predicted the adoption of a villainous moral identity rooted in moral inversion, whereby villains were recast as justified and not harmful and heroes as harmful. These moral reappraisals provided the psychological bridge between passion and violent endorsement, showing how OP can normalize violence by redefining its moral meaning. In contrast, HP predicted a heroic moral identity and conventional moral appraisals, reinforcing the view that heroes are good and villains are harmful and thereby offering protection against violent endorsement. Together, these findings underscore that violent extremism is not only a matter of ideology or circumstance but also of how passion reshapes moral self‐conceptions.

## AUTHOR CONTRIBUTIONS

**Jocelyn J. Bélanger:** Conceptualization; investigation; writing – original draft; methodology; validation; visualization; writing – review and editing; formal analysis; project administration; data curation; supervision; resources; software. **Daniel W. Snook:** Conceptualization; investigation; writing – original draft; methodology; writing – review and editing; validation. **Jais‐Adam Troian:** Conceptualization; investigation; writing – original draft; methodology; validation; writing – review and editing. **Manuel Moyano:** Conceptualization; investigation; writing – original draft; methodology; validation; writing – review and editing.

## CONFLICT OF INTEREST STATEMENT

The authors have no conflicts of interest to declare.

## Supporting information


Table S1.

## Data Availability

All data are available on the Open Science Framework (OSF) at the following link: https://osf.io/vamzt/?view_only=1c6964c7250448e3a3441862e656da59.
