# Supplementary material for: The role of moral identity in ideological obsession and violent extremism
Source: Br J Soc Psychol. 2026 Jun 18;65(3):e70106. doi: 10.1111/bjso.70106 (PMC13277983; doi:10.1111/bjso.70106)
Supplement: Supplementary file 1 — Table S1. [file BJSO-65-0-s001.docx]

**Supplementary File**

*The Role of Moral Identity in Ideological Obsession and Violent Extremism*

# Table of Contents

**Section 1.** Measures Used Across Studies

1.1 Study 1A — U.S. Democrats

1.2 Study 1B — U.S. Republicans

1.3 Study 2 — Environmental Cause

1.4 Study 3 — Black Lives Matter

1.5 Study 4 — U.S. Muslims

1.6 Study 5 — U.S. Republicans

1.7 Study 6 — Environmental Cause

**Section 2.** Factor Analyses for Study 6 (Tables S1–S14)

2.1 Harm Judgments (Tables S1–S4)

2.2 Justification Judgments (Tables S5–S8)

2.3 Identification with Hero and Villain (Table S9)

2.4 Dualistic Passion Scale (Table S10)

2.5 Commitment toward the Environmental Cause (Table S11)

2.6 Discriminant Validity Analyses (Tables S12–S14)

**Section 3.** Standardized Regression Weights — Main Models (Tables S15–S23)

**Section 4.** Standardized Regression Weights — Gender as Covariate (Tables S24–S30)

# Section 1. Measures Used Across Studies

This section reports the measures used in each of the six studies. Where measures are repeated across studies (e.g., the Dualistic Passion Scale, Moral Identity Picture Scale, Support for Political Violence), full text is provided at first appearance and referenced thereafter.

## 1.1 Study 1A — U.S. Democrats

### Ideological Passion

Response options (1–7):

- Not agree at all
- Very slightly agree
- Slightly agree
- Moderately agree
- Mostly agree
- Strongly agree
- Very strongly agree

While thinking of the Democratic Party, please indicate the extent to which you agree with each statement:

- My involvement in the Democratic Party is well integrated into my life.
- If I could, I would devote myself entirely to the Democratic Party.
- My involvement in the Democratic Party is in harmony with other things that are part of me.
- My involvement in the Democratic Party is so exciting that I sometimes lose control over it.
- I have the impression that my involvement in the Democratic Party controls me.
- My involvement in the Democratic Party is in harmony with the other activities in my life.
- I have difficulties controlling my urge to think about the Democratic Party.
- The new things I discover about the Democratic Party allow me to appreciate it even more.
- I have almost an obsessive feeling for the Democratic Party.
- My involvement in the Democratic Party reflects the qualities I like about myself.
- My involvement in the Democratic Party allows me to live a variety of experiences.
- The Democratic Party is the only thing that I can think of.
- I invest a lot of time in the Democratic Party.
- I like the Democratic Party.
- The Democratic Party is important to me.
- The Democratic Party is a passion for me.

*Scoring: Harmonious Passion = items 1, 3, 5, 6, 8, 10. Obsessive Passion = items 2, 4, 7, 9, 11, 12. Ideological Commitment = items 13, 14, 15, 16.*

### Moral Identity Picture Scale

Hero: Average of (1/Left, 2/Right, 5/Right, 6/Left, 9/Right, 10/Right, 13/Left, 16/Right)

Villain: Average of (1/Right, 3/Left, 5/Left, 7/Left, 9/Left, 11/Left, 13/Right, 14/Left)

Victim: Average of (3/Right, 4/Left, 7/Right, 8/Right, 11/Right, 12/Right, 14/Right, 15/Right)

Beneficiary: Average of (2/Left, 4/Right, 6/Right, 8/Left, 10/Left, 12/Left, 15/Left, 16/Left)

### Support for Political Violence

Response options (1–7):

- Not agree at all
- Very slightly agree
- Slightly agree
- Moderately agree
- Mostly agree
- Strongly agree
- Very strongly agree

For each statement below, indicate your level of agreement.

- Doing risky or illegal actions to help the Democratic Party win the next elections.
- Harassing supporters of the Republican Party.
- Physically attacking Republicans.
- Destroying signs that support the Republican Party.
- Using any means, even violent ones, to prevent the Republican Party from being elected.

## 1.2 Study 1B — U.S. Republicans

### Ideological Passion

Response options (1–7):

- Not agree at all
- Very slightly agree
- Slightly agree
- Moderately agree
- Mostly agree
- Strongly agree
- Very strongly agree

While thinking of the Republican Party, please indicate the extent to which you agree with each statement:

- My involvement in the Republican Party is well integrated into my life.
- If I could, I would devote myself entirely to the Republican Party.
- My involvement in the Republican Party is in harmony with other things that are part of me.
- My involvement in the Republican Party is so exciting that I sometimes lose control over it.
- I have the impression that my involvement in the Republican Party controls me.
- My involvement in the Republican Party is in harmony with the other activities in my life.
- I have difficulties controlling my urge to think about the Republican Party.
- The new things I discover about the Republican Party allow me to appreciate it even more.
- I have almost an obsessive feeling for the Republican Party.
- My involvement in the Republican Party reflects the qualities I like about myself.
- My involvement in the Republican Party allows me to live a variety of experiences.
- The Republican Party is the only thing that I can think of.
- I invest a lot of time in the Republican Party.
- I like the Republican Party.
- The Republican Party is important to me.
- The Republican Party is a passion for me.

*Scoring: Harmonious Passion = items 1, 3, 5, 6, 8, 10. Obsessive Passion = items 2, 4, 7, 9, 11, 12. Ideological Commitment = items 13, 14, 15, 16.*

### Moral Identity Picture Scale

*Same as in Study 1A.*

### Support for Political Violence

Response options (1–7):

- Not agree at all
- Very slightly agree
- Slightly agree
- Moderately agree
- Mostly agree
- Strongly agree
- Very strongly agree

For each statement below, please indicate your level of agreement:

- Doing risky or illegal actions to prevent the Democratic Party from winning the next elections.
- Harassing supporters of the Democratic Party.
- Physically attacking Democrats.
- Destroying signs that support the Democratic Party.
- Using any means, even violent ones, to prevent the Democratic Party from being elected.

## 1.3 Study 2 — Environmental Cause

### Harmonious Passion Prompt

Write about a time when your involvement in the environmental cause was in harmony with other things that are part of you and you felt that your involvement in the environmental cause allowed you to live a variety of experiences. Recall this time vividly and include as many details as you can to relive the experience. If this had never happened to you, imagine what such an event would feel like.

### Obsessive Passion Prompt

Write about a time when you had difficulties controlling your urge to get involved in the environmental cause and you felt that your involvement in the environmental cause was the only thing that really captivated you. Recall this time vividly and include as many details as you can to relive the experience. If this had never happened to you, imagine what such an event would feel like.

### Moral Identity Picture Scale

*Vignettes 1, 9, and 13 from the Moral Identity Picture Scale (see Study 1A).*

### Support for Political Violence

Response options (1–7):

- Not agree at all
- Very slightly agree
- Slightly agree
- Moderately agree
- Mostly agree
- Strongly agree
- Very strongly agree

For each statement below, please indicate your level of agreement:

- Joining a radical activist group to do risky or illegal actions in order to help the environmental cause.
- Forming a radical group to crack down on polluting businesses.
- Physically attacking a representative of a polluting factory.
- Appealing to acts of sabotage against installations that harm the environment.
- Using any means, even violent ones, to help the environmental cause.

## 1.4 Study 3 — Black Lives Matter

### Villain Identity Condition Prompt

Please write a brief fictional story with you as the lead character. In this story, you should describe a scene in which you harm another person. Try to write the story so that whoever reads it gets the impression that you are truly evil. The story should be realistic.

### Control Prompt

Please write a brief fictional story with you as the lead character. In this story, you should describe a scene in which you have lunch with another person. Try to write the story so that whoever reads it gets the impression of the lunch. The story should be realistic.

### Moral Identity Picture Scale

*Vignettes 1, 5, 9, and 13 from the Moral Identity Picture Scale (see Study 1A).*

### Support for Political Violence

Response options (1–7):

- Not agree at all
- Very slightly agree
- Slightly agree
- Moderately agree
- Mostly agree
- Strongly agree
- Very strongly agree

To what extent would you be willing to engage in the following behaviors to help your cause?

- Joining a radical activist group to do risky or illegal actions in order to help the Black Lives Matter movement.
- Burning down the car of a neo-Nazi.
- Physically attacking a neo-Nazi.
- Insulting a neo-Nazi.
- Using any means, even violent ones, to help the Black Lives Matter movement.

## 1.5 Study 4 — U.S. Muslims

### Ideological Passion

Response options (1–7):

- Not agree at all
- Very slightly agree
- Slightly agree
- Moderately agree
- Mostly agree
- Strongly agree
- Very strongly agree

While thinking of your relationship with Islam, please indicate the extent to which you agree with each statement:

- Islam is in harmony with the other activities in my life.
- I have difficulties controlling my urge to think about Islam.
- The new things I discover with Islam allow me to appreciate it even more.
- I have almost an obsessive feeling for Islam.
- Islam reflects the qualities I like about myself.
- Islam allows me to live a variety of experiences.
- Islam is the only thing that captivates me.
- Islam is well integrated into my life.
- If I could, I would devote myself entirely to Islam.
- Islam is in harmony with other things that are part of me.
- Islam is so exciting that I sometimes lose control over it.
- I have the impression that Islam controls me.
- I spend a lot of time thinking about Islam.
- I like Islam.
- Islam is important to me.
- Islam is a passion for me.

*Scoring: Harmonious Passion = items 1, 3, 5, 6, 8, 10. Obsessive Passion = items 2, 4, 7, 9, 11, 12. Ideological Commitment = items 13, 14, 15, 16.*

### Moral Identity Picture Scale

*Vignette 9 from the Moral Identity Picture Scale (see Study 1A).*

### Competence, Status, Warmth Scale

Response options (1–5):

- Strongly disagree
- Disagree
- Neither agree nor disagree
- Agree
- Strongly agree

Please rate the target on each of the following attributes:

- Competent
- Confident
- Independent
- Competitive
- Intelligent
- Tolerant
- Warm
- Good-natured
- Sincere
- Works in a prestigious job
- Economically successful
- Well-educated

*Scoring: Competence = items 1–5; Warmth = items 6–9; Status = items 10–12.*

### Support for Political Violence

Response options (1–7):

- Not agree at all
- Very slightly agree
- Slightly agree
- Moderately agree
- Mostly agree
- Strongly agree
- Very strongly agree

To what extent would you be willing to engage in the following behaviors for Islam?

- Doing risky or illegal actions to further the cause of Islam.
- Harassing people who ridicule or oppose Islam.
- Physically attacking people who ridicule or oppose Islam.
- Destroying signs that ridicule or oppose Islam.
- Using any means, even violent ones, to further the cause of Islam.

## 1.6 Study 5 — U.S. Republicans

### Ideological Passion

*Same items as in Study 1B.*

### Moral Identity Picture Scale

*Vignette 1 from the Moral Identity Picture Scale (see Study 1A).*

### Competence, Status, Warmth Scale

*Same as in Study 4.*

### Support for Political Violence

*Same items as in Study 1B.*

## 1.7 Study 6 — Environmental Cause

### Ideological Passion

Response options (1–7):

- Not agree at all
- Very slightly agree
- Slightly agree
- Moderately agree
- Mostly agree
- Strongly agree
- Very strongly agree

While thinking of the environmental cause, please indicate the extent to which you agree with each statement:

- My involvement in the environmental cause is in harmony with the other activities in my life.
- I have difficulties controlling my urge to being involved in the environmental cause.
- The new things that I discover about my involvement in the environmental cause allow me to appreciate it even more.
- I have almost an obsessive feeling for being involved in the environmental cause.
- My involvement in the environmental cause reflects the qualities I like about myself.
- My involvement in the environmental cause allows me to live a variety of experiences.
- My involvement in the environmental cause is the only thing that captivates me.
- My involvement in the environmental cause is well integrated in my life.
- If I could, I would only keep getting involved in the environmental cause.
- My involvement in the environmental cause is in harmony with other things that are part of me.
- My involvement in the environmental cause is so exciting that I sometimes lose control over it.
- I have the impression that my involvement in the environmental cause controls me.
- I spend a lot of time being involved in the environmental cause.
- I like the environmental cause.
- The environmental cause is important for me.
- The environmental cause is a passion for me.

*Scoring: Harmonious Passion = items 1, 3, 5, 6, 8, 10. Obsessive Passion = items 2, 4, 7, 9, 11, 12. Ideological Commitment = items 13, 14, 15, 16.*

### Moral Identity Picture Scale

*Vignettes 1, 5, 9, and 13 from the Moral Identity Picture Scale (see Study 1A).*

### Harm Items

Response options (1–4):

- Not at all
- Slightly
- Moderately
- Extremely

To what extent do you believe the actions of the person on the left and the person on the right…

*Person on the Left:*

- Are causing harm to others.
- Have negative consequences for innocent people.
- Are aggressive and violent.

*Person on the Right:*

- Are causing harm to others.
- Have negative consequences for innocent people.
- Are aggressive and violent.

### Justification Items

Response options (1–4):

- Not at all
- Slightly
- Moderately
- Extremely

To what extent do you believe the actions of the person on the left and the person on the right…

*Person on the Left:*

- Are justified given the circumstances.
- Are necessary to achieve a greater good.
- Are unavoidable to achieve an important objective.

*Person on the Right:*

- Are justified given the circumstances.
- Are necessary to achieve a greater good.
- Are unavoidable to achieve an important objective.

### Support for Political Violence

Response options (1–7):

- Not agree at all
- Very slightly agree
- Slightly agree
- Moderately agree
- Mostly agree
- Strongly agree
- Very strongly agree

For each statement below, please indicate your level of agreement:

- Joining a radical activist group to do risky or illegal actions in order to help the environmental cause.
- Forming a radical group to crack down on polluting businesses.
- Physically attack a polluting factory's representative.
- Appealing to acts of sabotage against installations that harm the environment.
- Using any means, even violent ones, to help the environmental cause.

# Section 2. Factor Analyses for Study 6

This section reports the exploratory factor analyses (EFAs) conducted in Study 6 (N = 236). All analyses used maximum likelihood extraction with direct oblimin rotation (δ = 0) in SPSS, with two factors extracted in each case unless otherwise specified. Loadings below .39 are suppressed from the pattern matrix in the SPSS output.

Items assessing harm and justification were evaluated separately for each of the four vignettes (Q17, Q21, Q25, Q29) because each vignette presented participants with a distinct pair of hero and villain characters. Because the four vignettes were designed as parallel operationalizations of the same underlying constructs, items were subsequently averaged across vignettes to form composite measures (Villain Harm, Hero Harm, Villain Justified, Hero Justified).

Additional EFAs were conducted on the eight identification items, the twelve-item Dualistic Passion Scale, the four-item commitment measure, and three discriminant-validity analyses pairing identification, passion, and radical action. Across all analyses, items loaded cleanly on their intended factors with no problematic cross-loadings, supporting the distinctiveness of the focal constructs. Note that the sign of factor correlations produced by oblimin rotation is arbitrary (determined by which factor the software labels as Factor 1); the magnitude is interpretable.

## 2.1 Harm Judgments

Within each vignette, the three harm items about the hero and the three harm items about the villain were submitted to a two-factor ML–oblimin EFA. In all four vignettes, the solution recovered a clean hero–villain structure, with items loading cleanly on their respective target factors.

**Table S1**

*Pattern Matrix — Harm Items, Vignette Q17 (N = 236)*

| **Item** | **Factor 1** | **Factor 2** |
| --- | --- | --- |
| Person on the Left — causing harm to others |  | .809 |
| Person on the Left — negative consequences for innocents |  | .646 |
| Person on the Left — aggressive and violent |  | .594 |
| Person on the Right — causing harm to others | .940 |  |
| Person on the Right — negative consequences for innocents | .605 |  |
| Person on the Right — aggressive and violent | .788 |  |

*Note.* Extraction: maximum likelihood. Rotation: oblimin with Kaiser normalization. Loadings < .39 suppressed. Eigenvalues (post-rotation): 2.15 / 1.66. Cumulative variance explained: 57.9%. Factor correlation: r = −.27.

**Table S2**

*Pattern Matrix — Harm Items, Vignette Q21 (N = 236)*

| **Item** | **Factor 1** | **Factor 2** |
| --- | --- | --- |
| Person on the Right — causing harm to others | 1.027 |  |
| Person on the Right — negative consequences for innocents | .855 |  |
| Person on the Right — aggressive and violent | .690 |  |
| Person on the Left — causing harm to others |  | .933 |
| Person on the Left — negative consequences for innocents |  | .955 |
| Person on the Left — aggressive and violent |  | .802 |

*Note.* Extraction: maximum likelihood. Rotation: oblimin with Kaiser normalization. Loadings < .39 suppressed. Eigenvalues (post-rotation): 3.44 / 3.54. Cumulative variance explained: 80.9%. Factor correlation: *r* = −.65.

**Table S3**

*Pattern Matrix — Harm Items, Vignette Q25 (N = 236)*

| **Item** | **Factor 1** | **Factor 2** |
| --- | --- | --- |
| Person on the Right — causing harm to others |  | .713 |
| Person on the Right — negative consequences for innocents |  | .772 |
| Person on the Right — aggressive and violent |  | .817 |
| Person on the Left — causing harm to others | .918 |  |
| Person on the Left — negative consequences for innocents | .937 |  |
| Person on the Left — aggressive and violent | .870 |  |

*Note.* Extraction: maximum likelihood. Rotation: oblimin with Kaiser normalization. Loadings < .39 suppressed. Eigenvalues (post-rotation): 2.81 / 2.19. Cumulative variance explained: 72.0%. Factor correlation: *r* = −.39.

**Table S4**

*Pattern Matrix — Harm Items, Vignette Q29 (N = 236)*

| **Item** | **Factor 1** | **Factor 2** |
| --- | --- | --- |
| Person on the Left — causing harm to others | −.989 |  |
| Person on the Left — negative consequences for innocents | −.718 |  |
| Person on the Left — aggressive and violent | −.745 |  |
| Person on the Right — causing harm to others |  | .913 |
| Person on the Right — negative consequences for innocents |  | .870 |
| Person on the Right — aggressive and violent |  | .800 |

*Note.* Extraction: maximum likelihood. Rotation: oblimin with Kaiser normalization. Loadings < .39 suppressed. Eigenvalues (post-rotation): 2.61 / 2.75. Cumulative variance explained: 71.8%. Factor correlation: *r* = .49 (software assigned opposite signs to the two factors in this vignette; the substantive hero–villain separation remains).

## 2.2 Justification Judgments

The same analytic procedure was applied to the three justification items (justified given the circumstances, necessary to achieve a greater good, unavoidable to achieve an important objective) about each target within each vignette. In all four vignettes, the two-factor solution recovered a clean hero–villain structure.

**Table S5**

*Pattern Matrix — Justification Items, Vignette Q17 (N = 236)*

| **Item** | **Factor 1** | **Factor 2** |
| --- | --- | --- |
| Person on the Left — justified given the circumstances | .848 |  |
| Person on the Left — necessary to achieve a greater good | .861 |  |
| Person on the Left — unavoidable to achieve an important objective | .780 |  |
| Person on the Right — justified given the circumstances |  | .728 |
| Person on the Right — necessary to achieve a greater good |  | .865 |
| Person on the Right — unavoidable to achieve an important objective |  | .759 |

*Note.* Extraction: maximum likelihood. Rotation: oblimin with Kaiser normalization. Loadings < .39 suppressed. Eigenvalues (post-rotation): 2.09 / 1.87. Cumulative variance explained: 65.7%. Factor correlation: *r* = −.05.

**Table S6**

*Pattern Matrix — Justification Items, Vignette Q21 (N = 236)*

| **Item** | **Factor 1** | **Factor 2** |
| --- | --- | --- |
| Person on the Right — justified given the circumstances | .855 |  |
| Person on the Right — necessary to achieve a greater good | .961 |  |
| Person on the Right — unavoidable to achieve an important objective | .760 |  |
| Person on the Left — justified given the circumstances |  | .825 |
| Person on the Left — necessary to achieve a greater good |  | .801 |
| Person on the Left — unavoidable to achieve an important objective |  | .706 |

*Note.* Extraction: maximum likelihood. Rotation: oblimin with Kaiser normalization. Loadings < .39 suppressed. Eigenvalues (post-rotation): 3.10 / 2.85. Cumulative variance explained: 69.5%. Factor correlation: *r* = −.64.

**Table S7**

*Pattern Matrix — Justification Items, Vignette Q25 (N = 236)*

| **Item** | **Factor 1** | **Factor 2** |
| --- | --- | --- |
| Person on the Right — justified given the circumstances | .792 |  |
| Person on the Right — necessary to achieve a greater good | .926 |  |
| Person on the Right — unavoidable to achieve an important objective | .774 |  |
| Person on the Left — justified given the circumstances |  | .658 |
| Person on the Left — necessary to achieve a greater good |  | .854 |
| Person on the Left — unavoidable to achieve an important objective |  | .665 |

*Note.* Extraction: maximum likelihood. Rotation: oblimin with Kaiser normalization. Loadings < .39 suppressed. Eigenvalues (post-rotation): 2.27 / 1.79. Cumulative variance explained: 62.9%. Factor correlation: *r* = −.26.

**Table S8**

*Pattern Matrix — Justification Items, Vignette Q29 (N = 236)*

| **Item** | **Factor 1** | **Factor 2** |
| --- | --- | --- |
| Person on the Left — justified given the circumstances | .846 |  |
| Person on the Left — necessary to achieve a greater good | .864 |  |
| Person on the Left — unavoidable to achieve an important objective | .745 |  |
| Person on the Right — justified given the circumstances |  | .657 |
| Person on the Right — necessary to achieve a greater good |  | .881 |
| Person on the Right — unavoidable to achieve an important objective |  | .677 |

*Note.* Extraction: maximum likelihood. Rotation: oblimin with Kaiser normalization. Loadings < .39 suppressed. Eigenvalues (post-rotation): 2.54 / 2.26. Cumulative variance explained: 63.0%. Factor correlation: r = −.51.

## 2.3 Identification with Hero and Villain

The eight identification items (one item per target per vignette, across all four vignettes) were submitted to a two-factor ML–oblimin EFA. The solution produced a clean hero–villain structure: items assessing identification with the hero target loaded on one factor and items assessing identification with the villain target loaded on the other.

**Table S9**

*Pattern Matrix — Identification with Hero and Villain Targets (8 items, N = 236)*

| **Item** | **Hero ID** | **Villain ID** |
| --- | --- | --- |
| Identification with hero (vignette 1) | .810 |  |
| Identification with hero (vignette 2) | .830 |  |
| Identification with hero (vignette 3) | .813 |  |
| Identification with hero (vignette 4) | .811 |  |
| Identification with villain (vignette 1) |  | .652 |
| Identification with villain (vignette 2) |  | .432 |
| Identification with villain (vignette 3) |  | .702 |
| Identification with villain (vignette 4) |  | .706 |

*Note.* Extraction: maximum likelihood. Rotation: oblimin with Kaiser normalization. Loadings < .39 suppressed. Eigenvalues (post-rotation): 2.89 / 1.65. Cumulative variance explained: 56.1%. Factor correlation: *r* = −.11. Item labels refer to identification with the morally heroic vs. morally villainous character across the four vignettes. Factor labels reflect the substantive content of the factors rather than arbitrary software labels.

## 2.4 Dualistic Passion Scale

The 12-item Dualistic Passion Scale (Vallerand et al., 2003) was submitted to a two-factor ML–oblimin EFA. The solution recovered the expected two-factor structure, with harmonious passion items (1, 3, 5, 6, 8, 10) loading on one factor and obsessive passion items (2, 4, 7, 9, 11, 12) loading on the other. The factor correlation of r = .37 is consistent with values typically reported in the literature for this scale.

**Table S10**

*Pattern Matrix — Dualistic Passion Scale (12 items, N = 236)*

| **Item** | **Obsessive** | **Harmonious** |
| --- | --- | --- |
| 1. Harmony with other activities in my life (HP) |  | .821 |
| 2. Difficulties controlling my urge to be involved (OP) | .735 |  |
| 3. New discoveries make me appreciate it more (HP) |  | .472 |
| 4. Almost obsessive feeling for being involved (OP) | .781 |  |
| 5. Reflects the qualities I like about myself (HP) |  | .749 |
| 6. Allows me to live a variety of experiences (HP) |  | .485 |
| 7. Only thing that captivates me (OP) | .908 |  |
| 8. Well integrated in my life (HP) |  | .681 |
| 9. If I could, I would only keep getting involved (OP) | .710 |  |
| 10. In harmony with other things that are part of me (HP) |  | .876 |
| 11. So exciting that I sometimes lose control over it (OP) | .916 |  |
| 12. Have the impression that it controls me (OP) | .963 |  |

*Note.* Extraction: maximum likelihood. Rotation: oblimin with Kaiser normalization. Loadings < .39 suppressed. HP = harmonious passion; OP = obsessive passion. Eigenvalues (post-rotation): 4.98 / 3.66. Cumulative variance explained: 62.9%. Factor correlation: *r* = .37. Factor labels reflect the substantive content of the factors.

## 2.5 Commitment toward the Environmental Cause

The four commitment items (Q12_13–Q12_16), which assess participants' commitment toward the environmental cause, were submitted to a single-factor ML EFA. Because only one factor was extracted, no rotation was performed. The solution yielded a single factor with an eigenvalue of 2.60, accounting for 64.93% of the variance. All four items loaded on the factor, with loadings ranging from .44 to .89. The three affective/identity-based items (Items 14–16) showed strong loadings (.69 to .89), while the behavioral involvement item (Item 13, “I spend a lot of time being involved in the environmental cause”) showed a weaker loading (.44) and a correspondingly lower communality (.19), likely reflecting its behavioral rather than attitudinal content.

**Table S11**

*Factor Matrix — Commitment toward the Environmental Cause (4 items, N = 236)*

| **Item** | **Factor 1** |
| --- | --- |
| 13. I spend a lot of time being involved in the environmental cause. | .435 |
| 14. I like the environmental cause. | .831 |
| 15. The environmental cause is important for me. | .894 |
| 16. The environmental cause is a passion for me. | .692 |

*Note.* Extraction: maximum likelihood. A single factor was specified; because only one factor was extracted, no rotation was performed. Initial eigenvalues: 2.60 / 0.87 / 0.31 / 0.23. Extraction sum of squared loadings: 2.16 (53.95% of variance). Initial total variance explained (eigenvalue > 1 criterion): 64.93%. The significant chi-square reflects residual covariance between items and is common for short scales where the single-factor model is constrained; the clear dominance of the first eigenvalue supports unidimensionality.

## 2.6 Discriminant Validity Analyses

Three additional two-factor EFAs were conducted to examine the discriminant validity of identification, dualistic passion, and radical action endorsement. Each analysis paired two constructs and tested whether their indicators would separate onto distinct factors.

### 2.6.1 Passion and Identification

The four hero-identification items were combined with the six obsessive passion items (Q12_2, 4, 7, 9, 11, 12). We focus on obsessive passion for this discriminant validity analysis because OP is the passion construct most theoretically relevant to identification with villain targets and support for political violence in our theoretical framework.

**Table S12**

*Pattern Matrix — Identification and Obsessive Passion (10 items, N = 236)*

| **Item** | **Obsessive Passion** | **Identification** |
| --- | --- | --- |
| Identification with hero (vignette 1) |  | .860 |
| Identification with hero (vignette 2) |  | .618 |
| Identification with hero (vignette 3) |  | .813 |
| Identification with hero (vignette 4) |  | .786 |
| 2. Difficulties controlling my urge to be involved (OP) | .591 |  |
| 4. Almost obsessive feeling for being involved (OP) | .762 |  |
| 7. Only thing that captivates me (OP) | .824 |  |
| 9. If I could, I would only keep getting involved (OP) | .881 |  |
| 11. So exciting that I sometimes lose control over it (OP) | .846 |  |
| 12. Have the impression that it controls me (OP) | .789 |  |

*Note.* Extraction: maximum likelihood. Rotation: oblimin with Kaiser normalization. Loadings < .39 suppressed. OP = obsessive passion. Eigenvalues (post-rotation): 5.60 / 4.91. Cumulative variance explained: 69.8%. Factor correlation: *r* = .68. Identification and obsessive passion emerged as empirically distinct factors, though they share substantial variance consistent with the theorized role of obsessive passion in shaping identification with villain targets.

### 2.6.2 Passion and Radical Action

The six obsessive passion items (Q12_2, 4, 7, 9, 11, 12) were combined with the five radical action items.

**Table S13**

*Pattern Matrix — Obsessive Passion and Radical Action (11 items, N = 236)*

| **Item** | **Radical Action** | **Obsessive Passion** |
| --- | --- | --- |
| 2. Difficulties controlling my urge to be involved (OP) |  | .506 |
| 4. Almost obsessive feeling for being involved (OP) |  | .699 |
| 7. Only thing that captivates me (OP) |  | .860 |
| 9. If I could, I would only keep getting involved (OP) |  | .832 |
| 11. So exciting that I sometimes lose control over it (OP) |  | .901 |
| 12. Have the impression that it controls me (OP) |  | .795 |
| Radical 1 — Joining a radical activist group | .896 |  |
| Radical 2 — Forming a radical group against polluters | .848 |  |
| Radical 3 — Physically attack a polluting factory's representative | .954 |  |
| Radical 4 — Appealing to acts of sabotage | .900 |  |
| Radical 5 — Using any means, even violent, to help the cause | .888 |  |

*Note.* Extraction: maximum likelihood. Rotation: oblimin with Kaiser normalization. Loadings < .39 suppressed. OP = obsessive passion. Eigenvalues (post-rotation): 6.81 / 6.57. Cumulative variance explained: 76.2%. Factor correlation: *r* = .75. Obsessive passion and radical action emerged as empirically distinct factors, though they share substantial variance consistent with the theorized role of obsessive passion in motivating radical action endorsement.

### 2.6.3 Identification and Radical Action

The four hero-identification items were combined with the five radical action items. In this analysis, the two factors were more strongly correlated (*r* = .81), indicating that identification with the environmental hero and endorsement of radical action share substantial variance in this sample. Despite this overlap, items loaded on their intended factors in the pattern matrix, consistent with the conceptual distinction between identity (how participants see themselves) and behavioral intention (what they would do). We retain the two constructs as separate composites in the main analyses.

**Table S14**

*Pattern Matrix — Identification and Radical Action (9 items, N = 236)*

| **Item** | **Radical Action** | **Identification** |
| --- | --- | --- |
| Identification with hero (vignette 1) |  | .776 |
| Identification with hero (vignette 2) | .482 | .426 |
| Identification with hero (vignette 3) |  | .723 |
| Identification with hero (vignette 4) |  | .926 |
| Radical 1 — Joining a radical activist group | .882 |  |
| Radical 2 — Forming a radical group against polluters | .822 |  |
| Radical 3 — Physically attack a polluting factory's representative | .947 |  |
| Radical 4 — Appealing to acts of sabotage | .971 |  |
| Radical 5 — Using any means, even violent, to help the cause | .917 |  |

*Note.* Extraction: maximum likelihood. Rotation: oblimin with Kaiser normalization. Loadings < .39 suppressed. Eigenvalues (post-rotation): 6.20 / 5.44. Cumulative variance explained: 77.2%. Factor correlation: *r* = .81. Identification and radical action items load on distinct factors but share substantial variance, consistent with the conceptual overlap between identifying with a cause and endorsing action on its behalf.

## 2.7 Summary

Across all exploratory factor analyses, items loaded cleanly on their intended factors with minimal cross-loadings, supporting the distinctiveness of the focal constructs. The within-vignette analyses of harm and justification items established that hero-directed and villain-directed judgments form coherent, target-specific factors within each vignette, justifying the creation of cross-vignette composites (Villain Harm, Hero Harm, Villain Justified, Hero Justified). The Dualistic Passion Scale replicated its well-established two-factor structure. Discriminant-validity analyses confirmed that identification, obsessive passion, and radical action are empirically distinguishable — items loaded on their intended factors in every pattern matrix. The substantial factor correlations among these constructs (*r*s ranging from .68 to .81) are consistent with the theoretical framework: obsessive passion is expected to shape identification with villain targets and motivate endorsement of radical action, so shared variance among these measures reflects the theorized mechanism rather than a measurement problem.

# Section 3. Standardized Regression Weights — Main Models

This section reports the full standardized regression weights for the path models presented in the main text of each study. Models were estimated in AMOS using full information maximum likelihood (FIML). Indirect effects, where reported in the main text, were computed using bias-corrected bootstrap confidence intervals (5,000 resamples).

**Table S15**

*Standardized Regression Weights — Study 1A (U.S. Democrats)*

| **Path** | **β** | **S.E.** | **C.R.** | **p** |
| --- | --- | --- | --- | --- |
| Hero ← OP | −.088 | .082 | −1.072 | .284 |
| Hero ← HP | .106 | .106 | 1.000 | .317 |
| Hero ← Ideological commitment | .329 | .115 | 2.876 | .004 ** |
| Villain ← OP | .438 | .065 | 6.753 | <.001 *** |
| Villain ← HP | −.159 | .084 | −1.904 | .057 |
| Villain ← Ideological commitment | −.213 | .090 | −2.368 | .018 * |
| Victim ← OP | .214 | .087 | 2.476 | .013 * |
| Victim ← HP | .000 | .112 | −.002 | .999 |
| Victim ← Ideological commitment | −.068 | .120 | −.567 | .570 |
| Support for political violence ← Hero | −.075 | .047 | −1.595 | .111 |
| Support for political violence ← Villain | .257 | .060 | 4.298 | <.001 *** |
| Support for political violence ← Victim | .014 | .045 | .303 | .762 |
| Support for political violence ← OP | .206 | .059 | 3.473 | <.001 *** |
| Support for political violence ← Ideological commitment | −.009 | .060 | −.155 | .877 |

*Note.* Standardized coefficients (β) are reported. * p < .05. ** p < .01. *** p < .001. OP = obsessive passion; HP = harmonious passion.

**Table S16**

*Standardized Regression Weights — Study 1B (U.S. Republicans)*

| **Path** | **β** | **S.E.** | **C.R.** | **p** |
| --- | --- | --- | --- | --- |
| Hero ← OP | −.188 | .081 | −2.326 | .020 * |
| Hero ← HP | .215 | .099 | 2.181 | .029 * |
| Hero ← Ideological commitment | .041 | .107 | .381 | .703 |
| Villain ← OP | .321 | .087 | 3.702 | <.001 *** |
| Villain ← HP | −.102 | .106 | −.967 | .334 |
| Villain ← Ideological commitment | .091 | .115 | .794 | .427 |
| Victim ← OP | .142 | .083 | 1.720 | .085 |
| Victim ← HP | −.030 | .101 | −.296 | .767 |
| Victim ← Ideological commitment | .072 | .109 | .658 | .510 |
| Support for political violence ← Hero | −.036 | .057 | −.642 | .521 |
| Support for political violence ← Villain | .132 | .052 | 2.568 | .010 ** |
| Support for political violence ← Victim | −.014 | .055 | −.247 | .805 |
| Support for political violence ← OP | .447 | .066 | 6.742 | <.001 *** |
| Support for political violence ← Ideological commitment | −.053 | .065 | −.812 | .417 |

*Note.* Standardized coefficients (β) are reported. * p < .05. ** p < .01. *** p < .001. OP = obsessive passion; HP = harmonious passion.

**Table S17**

*Standardized Regression Weights — Study 2 (Environmental Cause)*

| **Path** | **β** | **S.E.** | **C.R.** | **p** |
| --- | --- | --- | --- | --- |
| Hero ← Experimental condition | −.099 | .113 | −.875 | .381 |
| Villain ← Experimental condition | .295 | .112 | 2.644 | .008 ** |
| Support for political violence ← Hero | .001 | .054 | .020 | .984 |
| Support for political violence ← Villain | .310 | .054 | 5.750 | <.001 *** |

*Note.* Standardized coefficients (β) are reported. ** p < .01. *** p < .001. Experimental condition: 0 = harmonious passion, 1 = obsessive passion.

**Table S18**

*Standardized Regression Weights — Study 3 (Black Lives Matter): Alternative Mediation Model*

| **Path** | **β** | **S.E.** | **C.R.** | **p** |
| --- | --- | --- | --- | --- |
| OP ← Experimental condition | .037 | .075 | .489 | .625 |
| HP ← Experimental condition | −.010 | .075 | −.130 | .897 |
| Support for political violence ← OP | .405 | .083 | 4.890 | <.001 *** |
| Support for political violence ← HP | .101 | .083 | 1.226 | .220 |

*Note.* Standardized coefficients (β) are reported. *** p < .001. Experimental condition: 0 = control condition, 1 = villain condition. This model tests an alternative mediation account in which the moral-identity manipulation predicts support for political violence indirectly through obsessive (OP) and harmonious (HP) passion. Neither indirect effect was significant.

**Table S19**

*Standardized Regression Weights — Study 4 (U.S. Muslims): Relative Identification (Villain vs. Hero)*

| **Path** | **β** | **S.E.** | **C.R.** | **p** |
| --- | --- | --- | --- | --- |
| Competence (evil − hero) ← OP | −.136 | .108 | −1.256 | .209 |
| Competence (evil − hero) ← HP | .004 | .154 | .024 | .981 |
| Competence (evil − hero) ← Ideological commitment | −.263 | .165 | −1.591 | .112 |
| Warmth (evil − hero) ← OP | .621 | .112 | 5.522 | <.001 *** |
| Warmth (evil − hero) ← HP | −.359 | .160 | −2.240 | .025 * |
| Warmth (evil − hero) ← Ideological commitment | −.384 | .172 | −2.232 | .026 * |
| Status (evil − hero) ← OP | −.067 | .112 | −.600 | .549 |
| Status (evil − hero) ← HP | .004 | .160 | .024 | .981 |
| Status (evil − hero) ← Ideological commitment | −.251 | .172 | −1.465 | .143 |
| Villain ID (evil − hero) ← Competence (evil − hero) | .077 | .064 | 1.197 | .231 |
| Villain ID (evil − hero) ← Warmth (evil − hero) | .291 | .049 | 5.975 | <.001 *** |
| Villain ID (evil − hero) ← Status (evil − hero) | .132 | .061 | 2.148 | .032 * |

*Note.* Standardized coefficients (β) are reported. * p < .05. ** p < .01. *** p < .001. OP = obsessive passion; HP = harmonious passion.

**Table S20**

*Standardized Regression Weights — Study 4 (U.S. Muslims): Absolute Identification (The Appeal of Villains)*

| **Path** | **β** | **S.E.** | **C.R.** | **p** |
| --- | --- | --- | --- | --- |
| Competence (villain) ← OP | −.087 | .070 | −1.246 | .213 |
| Competence (villain) ← HP | .115 | .100 | 1.154 | .248 |
| Competence (villain) ← Ideological commitment | −.058 | .107 | −.544 | .586 |
| Warmth (villain) ← OP | .395 | .066 | 5.979 | <.001 *** |
| Warmth (villain) ← HP | −.084 | .094 | −.898 | .369 |
| Warmth (villain) ← Ideological commitment | −.164 | .101 | −1.622 | .105 |
| Status (villain) ← OP | .196 | .069 | 2.827 | .005 ** |
| Status (villain) ← HP | .014 | .099 | .146 | .884 |
| Status (villain) ← Ideological commitment | −.132 | .106 | −1.247 | .212 |
| Villain ID ← Competence (villain) | .021 | .053 | .398 | .691 |
| Villain ID ← Warmth (villain) | .535 | .060 | 8.890 | <.001 *** |
| Villain ID ← Status (villain) | .047 | .058 | .809 | .418 |
| Villain ID ← OP | .125 | .050 | 2.486 | .013 * |
| Support for political violence ← Villain ID | 1.191 | .156 | 7.635 | <.001 *** |
| Support for political violence ← OP | .340 | .109 | 3.112 | .002 ** |
| Support for political violence ← HP | −.121 | .125 | −.974 | .330 |
| Support for political violence ← Ideological commitment | .214 | .134 | 1.594 | .111 |

*Note.* Standardized coefficients (β) are reported. * p < .05. ** p < .01. *** p < .001. OP = obsessive passion; HP = harmonious passion.

**Table S21**

*Standardized Regression Weights — Study 5 (U.S. Republicans): Relative Identification (Villain vs. Hero)*

| **Path** | **β** | **S.E.** | **C.R.** | **p** |
| --- | --- | --- | --- | --- |
| Competence (evil − hero) ← OP | −.178 | .073 | −2.445 | .014 * |
| Competence (evil − hero) ← HP | .121 | .094 | 1.287 | .198 |
| Competence (evil − hero) ← Ideological commitment | −.134 | .103 | −1.298 | .194 |
| Warmth (evil − hero) ← OP | .207 | .073 | 2.831 | .005 ** |
| Warmth (evil − hero) ← HP | −.005 | .094 | −.055 | .956 |
| Warmth (evil − hero) ← Ideological commitment | −.215 | .104 | −2.067 | .039 * |
| Status (evil − hero) ← OP | −.096 | .073 | −1.311 | .190 |
| Status (evil − hero) ← HP | .086 | .094 | .915 | .360 |
| Status (evil − hero) ← Ideological commitment | −.194 | .103 | −1.870 | .062 |
| Villain ID (evil − hero) ← Competence (evil − hero) | .127 | .070 | 1.823 | .068 |
| Villain ID (evil − hero) ← Warmth (evil − hero) | .404 | .057 | 7.029 | <.001 *** |
| Villain ID (evil − hero) ← Status (evil − hero) | .018 | .067 | .263 | .793 |

*Note.* Standardized coefficients (β) are reported. * p < .05. ** p < .01. *** p < .001. OP = obsessive passion; HP = harmonious passion.

**Table S22**

*Standardized Regression Weights — Study 5 (U.S. Republicans): Absolute Identification (The Appeal of Villains)*

| **Path** | **β** | **S.E.** | **C.R.** | **p** |
| --- | --- | --- | --- | --- |
| Competence (villain) ← OP | −.202 | .073 | −2.777 | .005 ** |
| Competence (villain) ← HP | .165 | .094 | 1.760 | .078 |
| Competence (villain) ← Ideological commitment | −.080 | .103 | −.775 | .438 |
| Warmth (villain) ← OP | .162 | .074 | 2.202 | .028 * |
| Warmth (villain) ← HP | .069 | .095 | .727 | .467 |
| Warmth (villain) ← Ideological commitment | −.193 | .105 | −1.839 | .066 |
| Status (villain) ← OP | −.035 | .074 | −.477 | .633 |
| Status (villain) ← HP | .119 | .095 | 1.246 | .213 |
| Status (villain) ← Ideological commitment | −.174 | .105 | −1.668 | .095 |
| Villain ID ← Competence (villain) | .074 | .073 | 1.018 | .309 |
| Villain ID ← Warmth (villain) | .257 | .061 | 4.209 | <.001 *** |
| Villain ID ← Status (villain) | .086 | .070 | 1.221 | .222 |
| Villain ID ← OP | .174 | .055 | 3.156 | .002 ** |
| Support for political violence ← Villain ID | .080 | .045 | 1.804 | .071 |
| Support for political violence ← OP | .245 | .057 | 4.328 | <.001 *** |
| Support for political violence ← HP | −.048 | .072 | −.667 | .505 |
| Support for political violence ← Ideological commitment | .080 | .080 | 1.005 | .315 |

*Note.* Standardized coefficients (β) are reported. * p < .05. ** p < .01. *** p < .001. OP = obsessive passion; HP = harmonious passion.

**Table S23**

*Standardized Regression Weights — Study 6 (Environmental Cause)*

| **Path** | **β** | **S.E.** | **C.R.** | **p** |
| --- | --- | --- | --- | --- |
| Villain ID ← OP | .743 | .054 | 13.820 | <.001 *** |
| Villain ID ← HP | −.193 | .077 | −2.507 | .012 * |
| Villain ID ← Ideological commitment | .079 | .082 | .960 | .337 |
| Hero ID ← OP | .027 | .073 | .377 | .706 |
| Hero ID ← HP | .357 | .104 | 3.433 | <.001 *** |
| Hero ID ← Ideological commitment | −.055 | .111 | −.492 | .622 |
| Villain Harm ← Villain ID | −.285 | .063 | −4.548 | <.001 *** |
| Villain Harm ← Hero ID | .313 | .061 | 5.106 | <.001 *** |
| Villain Harm ← Ideological commitment | .070 | .064 | 1.084 | .279 |
| Hero Harm ← Villain ID | .520 | .057 | 9.114 | <.001 *** |
| Hero Harm ← Hero ID | .054 | .044 | 1.225 | .221 |
| Hero Harm ← OP | .351 | .058 | 6.080 | <.001 *** |
| Hero Harm ← Ideological commitment | −.108 | .049 | −2.223 | .026 * |
| Villain Justified ← Villain ID | .569 | .051 | 11.119 | <.001 *** |
| Villain Justified ← Hero ID | −.042 | .038 | −1.090 | .276 |
| Villain Justified ← OP | .384 | .055 | 6.989 | <.001 *** |
| Villain Justified ← Ideological commitment | −.153 | .043 | −3.610 | <.001 *** |
| Hero Justified ← Villain ID | −.083 | .063 | −1.320 | .187 |
| Hero Justified ← Hero ID | .408 | .062 | 6.620 | <.001 *** |
| Hero Justified ← Ideological commitment | .067 | .065 | 1.039 | .299 |
| Support for political violence ← Villain ID | .736 | .116 | 6.343 | <.001 *** |
| Support for political violence ← Villain Harm | −.135 | .080 | −1.688 | .091 |
| Support for political violence ← Villain Justified | .284 | .123 | 2.305 | .021 * |
| Support for political violence ← Hero Harm | .386 | .115 | 3.342 | <.001 *** |
| Support for political violence ← Hero Justified | .100 | .074 | 1.358 | .175 |
| Support for political violence ← OP | .466 | .113 | 4.115 | <.001 *** |
| Support for political violence ← HP | .181 | .106 | 1.702 | .089 |
| Support for political violence ← Ideological commitment | .052 | .113 | .455 | .649 |

*Note.* Standardized coefficients (β) are reported. * p < .05. ** p < .01. *** p < .001. OP = obsessive passion; HP = harmonious passion.

# Section 4. Standardized Regression Weights — Robustness Check with Gender as Covariate

As a robustness check, the path models from Section 3 were re-estimated with participant gender included as a covariate. Results across studies are substantively unchanged from the main models reported in Section 3. Tables below report only the studies for which gender was available; Studies 2 and 3 used experimental designs with random assignment and are not re-estimated here.

**Table S24**

*Standardized Regression Weights with Gender Covariate — Study 1A (U.S. Democrats)*

| **Path** | **β** | **S.E.** | **C.R.** | **p** |
| --- | --- | --- | --- | --- |
| Hero ← OP | −.099 | .082 | −1.204 | .229 |
| Hero ← HP | .129 | .107 | 1.203 | .229 |
| Hero ← Ideological commitment | .313 | .115 | 2.730 | .006 ** |
| Hero ← Gender | −.160 | .128 | −1.253 | .210 |
| Villain ← OP | .430 | .065 | 6.615 | <.001 *** |
| Villain ← HP | −.143 | .085 | −1.688 | .091 |
| Villain ← Ideological commitment | −.225 | .090 | −2.488 | .013 * |
| Villain ← Gender | −.115 | .101 | −1.145 | .252 |
| Victim ← OP | .236 | .086 | 2.745 | .006 ** |
| Victim ← HP | −.045 | .112 | −.402 | .688 |
| Victim ← Ideological commitment | −.037 | .120 | −.311 | .756 |
| Victim ← Gender | .313 | .133 | 2.349 | .019 * |
| Support for political violence ← Hero | −.087 | .047 | −1.853 | .064 |
| Support for political violence ← Villain | .238 | .060 | 3.974 | <.001 *** |
| Support for political violence ← Victim | .033 | .046 | .723 | .470 |
| Support for political violence ← OP | .199 | .059 | 3.375 | <.001 *** |
| Support for political violence ← Ideological commitment | −.008 | .059 | −.137 | .891 |
| Support for political violence ← Gender | −.179 | .084 | −2.119 | .034 * |

*Note.* Standardized coefficients (β) are reported. * p < .05. ** p < .01. *** p < .001. OP = obsessive passion; HP = harmonious passion. Gender coded 0 = men, 1 = women, 2 = other.

**Table S25**

*Standardized Regression Weights with Gender Covariate — Study 1B (U.S. Republicans)*

| **Path** | **β** | **S.E.** | **C.R.** | **p** |
| --- | --- | --- | --- | --- |
| Hero ← OP | −.150 | .082 | −1.837 | .066 |
| Hero ← HP | .189 | .099 | 1.918 | .055 |
| Hero ← Ideological commitment | .032 | .106 | .302 | .763 |
| Hero ← Gender | .294 | .136 | 2.172 | .030 * |
| Villain ← OP | .259 | .087 | 2.985 | .003 ** |
| Villain ← HP | −.058 | .105 | −.550 | .582 |
| Villain ← Ideological commitment | .102 | .113 | .909 | .363 |
| Villain ← Gender | −.482 | .144 | −3.357 | <.001 *** |
| Victim ← OP | .152 | .085 | 1.797 | .072 |
| Victim ← HP | −.032 | .102 | −.309 | .758 |
| Victim ← Ideological commitment | .064 | .110 | .581 | .561 |
| Victim ← Gender | .093 | .140 | .662 | .508 |
| Support for political violence ← Hero | −.021 | .056 | −.382 | .702 |
| Support for political violence ← Villain | .101 | .052 | 1.953 | .051 |
| Support for political violence ← Victim | −.006 | .055 | −.104 | .917 |
| Support for political violence ← OP | .418 | .066 | 6.359 | <.001 *** |
| Support for political violence ← Ideological commitment | −.025 | .064 | −.382 | .703 |
| Support for political violence ← Gender | −.336 | .109 | −3.073 | .002 ** |

*Note.* Standardized coefficients (β) are reported. * p < .05. ** p < .01. *** p < .001. OP = obsessive passion; HP = harmonious passion. Gender coded 0 = men, 1 = women, 2 = other. One participant did not report their gender and was excluded from the analysis.

**Table S26**

*Standardized Regression Weights with Gender Covariate — Study 4 (U.S. Muslims): Relative Identification*

| **Path** | **β** | **S.E.** | **C.R.** | **p** |
| --- | --- | --- | --- | --- |
| Competence (evil − hero) ← OP | −.134 | .108 | −1.241 | .215 |
| Competence (evil − hero) ← HP | .007 | .154 | .043 | .965 |
| Competence (evil − hero) ← Ideological commitment | −.270 | .165 | −1.635 | .102 |
| Competence (evil − hero) ← Gender | −.236 | .188 | −1.255 | .209 |
| Warmth (evil − hero) ← OP | .624 | .112 | 5.572 | <.001 *** |
| Warmth (evil − hero) ← HP | −.355 | .159 | −2.225 | .026 * |
| Warmth (evil − hero) ← Ideological commitment | −.393 | .171 | −2.293 | .022 * |
| Warmth (evil − hero) ← Gender | −.319 | .195 | −1.633 | .102 |
| Status (evil − hero) ← OP | −.066 | .112 | −.589 | .556 |
| Status (evil − hero) ← HP | .006 | .159 | .036 | .971 |
| Status (evil − hero) ← Ideological commitment | −.256 | .171 | −1.491 | .136 |
| Status (evil − hero) ← Gender | −.154 | .195 | −.787 | .432 |
| Villain ID (evil − hero) ← Competence (evil − hero) | .077 | .064 | 1.203 | .229 |
| Villain ID (evil − hero) ← Warmth (evil − hero) | .292 | .049 | 5.977 | <.001 *** |
| Villain ID (evil − hero) ← Status (evil − hero) | .132 | .062 | 2.142 | .032 * |
| Villain ID (evil − hero) ← Gender | .025 | .135 | .188 | .851 |

*Note.* Standardized coefficients (β) are reported. * p < .05. ** p < .01. *** p < .001. OP = obsessive passion; HP = harmonious passion. Gender coded 0 = men, 1 = women, 2 = other.

**Table S27**

*Standardized Regression Weights with Gender Covariate — Study 4 (U.S. Muslims): Absolute Identification*

| **Path** | **β** | **S.E.** | **C.R.** | **p** |
| --- | --- | --- | --- | --- |
| Competence (villain) ← OP | −.053 | .043 | −1.230 | .219 |
| Competence (villain) ← HP | .097 | .082 | 1.179 | .238 |
| Competence (villain) ← Ideological commitment | −.049 | .083 | −.588 | .557 |
| Competence (villain) ← Gender | −.166 | .124 | −1.339 | .181 |
| Warmth (villain) ← OP | .223 | .037 | 6.046 | <.001 *** |
| Warmth (villain) ← HP | −.061 | .070 | −.873 | .383 |
| Warmth (villain) ← Ideological commitment | −.120 | .071 | −1.693 | .090 |
| Warmth (villain) ← Gender | −.203 | .106 | −1.916 | .055 |
| Status (villain) ← OP | .123 | .043 | 2.841 | .005 ** |
| Status (villain) ← HP | .013 | .083 | .157 | .875 |
| Status (villain) ← Ideological commitment | −.106 | .083 | −1.271 | .204 |
| Status (villain) ← Gender | −.091 | .125 | −.732 | .464 |
| Villain ID ← Competence (villain) | .017 | .037 | .466 | .641 |
| Villain ID ← Warmth (villain) | .414 | .046 | 8.999 | <.001 *** |
| Villain ID ← Status (villain) | .029 | .040 | .738 | .461 |
| Villain ID ← OP | .053 | .022 | 2.471 | .013 * |
| Villain ID ← Gender | .082 | .069 | 1.191 | .234 |
| Support for political violence ← Villain ID | 1.685 | .219 | 7.692 | <.001 *** |
| Support for political violence ← OP | .204 | .066 | 3.081 | .002 ** |
| Support for political violence ← HP | −.100 | .101 | −.986 | .324 |
| Support for political violence ← Ideological commitment | .166 | .102 | 1.628 | .103 |
| Support for political violence ← Gender | .036 | .174 | .207 | .836 |

*Note.* Standardized coefficients (β) are reported. * p < .05. ** p < .01. *** p < .001. OP = obsessive passion; HP = harmonious passion. Gender coded 0 = men, 1 = women, 2 = other.

**Table S28**

*Standardized Regression Weights with Gender Covariate — Study 5 (U.S. Republicans): Relative Identification*

| **Path** | **β** | **S.E.** | **C.R.** | **p** |
| --- | --- | --- | --- | --- |
| Competence (evil − hero) ← OP | −.179 | .072 | −2.476 | .013 * |
| Competence (evil − hero) ← HP | .126 | .094 | 1.352 | .176 |
| Competence (evil − hero) ← Ideological commitment | −.136 | .103 | −1.328 | .184 |
| Competence (evil − hero) ← Gender | −.204 | .114 | −1.787 | .074 |
| Warmth (evil − hero) ← OP | .205 | .072 | 2.833 | .005 ** |
| Warmth (evil − hero) ← HP | .002 | .094 | .019 | .985 |
| Warmth (evil − hero) ← Ideological commitment | −.218 | .103 | −2.113 | .035 * |
| Warmth (evil − hero) ← Gender | −.257 | .114 | −2.253 | .024 * |
| Status (evil − hero) ← OP | −.097 | .073 | −1.329 | .184 |
| Status (evil − hero) ← HP | .091 | .094 | .964 | .335 |
| Status (evil − hero) ← Ideological commitment | −.195 | .103 | −1.894 | .058 |
| Status (evil − hero) ← Gender | −.160 | .115 | −1.398 | .162 |
| Villain ID (evil − hero) ← Competence (evil − hero) | .126 | .070 | 1.801 | .072 |
| Villain ID (evil − hero) ← Warmth (evil − hero) | .400 | .058 | 6.939 | <.001 *** |
| Villain ID (evil − hero) ← Status (evil − hero) | .017 | .067 | .258 | .797 |
| Villain ID (evil − hero) ← Gender | −.070 | .103 | −.680 | .497 |

*Note.* Standardized coefficients (β) are reported. * p < .05. ** p < .01. *** p < .001. OP = obsessive passion; HP = harmonious passion. Gender coded 0 = men, 1 = women, 2 = other.

**Table S29**

*Standardized Regression Weights with Gender Covariate — Study 5 (U.S. Republicans): Absolute Identification*

| **Path** | **β** | **S.E.** | **C.R.** | **p** |
| --- | --- | --- | --- | --- |
| Competence (villain) ← OP | −.202 | .073 | −2.780 | .005 ** |
| Competence (villain) ← HP | .166 | .094 | 1.768 | .077 |
| Competence (villain) ← Ideological commitment | −.080 | .103 | −.779 | .436 |
| Competence (villain) ← Gender | −.030 | .115 | −.265 | .791 |
| Warmth (villain) ← OP | .161 | .073 | 2.197 | .028 * |
| Warmth (villain) ← HP | .075 | .095 | .793 | .428 |
| Warmth (villain) ← Ideological commitment | −.195 | .104 | −1.873 | .061 |
| Warmth (villain) ← Gender | −.215 | .116 | −1.856 | .063 |
| Status (villain) ← OP | −.035 | .074 | −.480 | .631 |
| Status (villain) ← HP | .119 | .095 | 1.255 | .210 |
| Status (villain) ← Ideological commitment | −.175 | .105 | −1.672 | .094 |
| Status (villain) ← Gender | −.033 | .116 | −.280 | .779 |
| Villain ID ← Competence (villain) | .076 | .073 | 1.042 | .298 |
| Villain ID ← Warmth (villain) | .252 | .061 | 4.097 | <.001 *** |
| Villain ID ← Status (villain) | .086 | .070 | 1.229 | .219 |
| Villain ID ← OP | .175 | .055 | 3.173 | .002 ** |
| Villain ID ← Gender | −.086 | .108 | −.794 | .427 |
| Support for political violence ← Villain ID | .071 | .044 | 1.617 | .106 |
| Support for political violence ← OP | .245 | .056 | 4.384 | <.001 *** |
| Support for political violence ← HP | −.041 | .071 | −.579 | .562 |
| Support for political violence ← Ideological commitment | .077 | .079 | .980 | .327 |
| Support for political violence ← Gender | −.240 | .087 | −2.745 | .006 ** |

*Note.* Standardized coefficients (β) are reported. * p < .05. ** p < .01. *** p < .001. OP = obsessive passion; HP = harmonious passion. Gender coded 0 = men, 1 = women, 2 = other.

**Table S30**

*Standardized Regression Weights with Gender Covariate — Study 6 (Environmental Cause)*

| **Path** | **β** | **S.E.** | **C.R.** | **p** |
| --- | --- | --- | --- | --- |
| Villain ID ← OP | .744 | .054 | 13.824 | <.001 *** |
| Villain ID ← HP | −.191 | .077 | −2.472 | .013 * |
| Villain ID ← Ideological commitment | .077 | .082 | .934 | .350 |
| Villain ID ← Gender | −.032 | .087 | −.363 | .716 |
| Hero ID ← OP | .023 | .073 | .320 | .749 |
| Hero ID ← HP | .348 | .104 | 3.346 | <.001 *** |
| Hero ID ← Ideological commitment | −.047 | .111 | −.419 | .675 |
| Hero ID ← Gender | .130 | .117 | 1.107 | .268 |
| Villain Harm ← Villain ID | −.285 | .063 | −4.544 | <.001 *** |
| Villain Harm ← Hero ID | .314 | .062 | 5.095 | <.001 *** |
| Villain Harm ← Ideological commitment | .070 | .064 | 1.082 | .279 |
| Villain Harm ← Gender | −.013 | .111 | −.114 | .909 |
| Hero Harm ← Villain ID | .520 | .057 | 9.157 | <.001 *** |
| Hero Harm ← Hero ID | .050 | .044 | 1.127 | .260 |
| Hero Harm ← OP | .347 | .057 | 6.043 | <.001 *** |
| Hero Harm ← Ideological commitment | −.107 | .049 | −2.195 | .028 * |
| Hero Harm ← Gender | .094 | .079 | 1.201 | .230 |
| Villain Justified ← Villain ID | .570 | .051 | 11.152 | <.001 *** |
| Villain Justified ← Hero ID | −.045 | .038 | −1.184 | .237 |
| Villain Justified ← OP | .381 | .055 | 6.956 | <.001 *** |
| Villain Justified ← Ideological commitment | −.152 | .042 | −3.588 | <.001 *** |
| Villain Justified ← Gender | .077 | .068 | 1.141 | .254 |
| Hero Justified ← Villain ID | −.082 | .063 | −1.307 | .191 |
| Hero Justified ← Hero ID | .411 | .062 | 6.644 | <.001 *** |
| Hero Justified ← Ideological commitment | .067 | .065 | 1.033 | .302 |
| Hero Justified ← Gender | −.059 | .112 | −.528 | .597 |
| Support for political violence ← Villain ID | .719 | .116 | 6.191 | <.001 *** |
| Support for political violence ← Villain Harm | −.126 | .081 | −1.559 | .119 |
| Support for political violence ← Villain Justified | .268 | .124 | 2.164 | .030 * |
| Support for political violence ← Hero Harm | .394 | .116 | 3.379 | <.001 *** |
| Support for political violence ← Hero Justified | .114 | .074 | 1.542 | .123 |
| Support for political violence ← OP | .492 | .114 | 4.331 | <.001 *** |
| Support for political violence ← Ideological commitment | .188 | .076 | 2.461 | .014 * |
| Support for political violence ← Gender | −.037 | .120 | −.310 | .756 |

*Note.* Standardized coefficients (β) are reported. * p < .05. ** p < .01. *** p < .001. OP = obsessive passion; HP = harmonious passion. Gender coded 1 = men, 2 = women, 3 = non-binary/third gender, 4 = prefer not to say.
